# Supporting Informal Dementia Caregivers Through an iSupport Web-Based Primary Health Care Intervention: Hybrid Effectiveness-Implementation Mixed Methods Study

**DOI:** 10.2196/77688

**Published:** 2025-09-09

**Authors:** Shasha Yuan, Jingbin Zhang, Zheng Wang, Huali Wang, Mengmeng Xia

**Affiliations:** 1 Institute of Medical Information/Medical Library Chinese Academy of Medical Sciences & Peking Union Medical College Beijing China; 2 Dementia Care and Research Center Peking University Institute of Mental Health (Sixth Hospital) Beijing China

**Keywords:** informal dementia caregivers, primary health care, iSupport program, implementation science, cluster randomized controlled trial

## Abstract

**Background:**

Informal caregivers of home-dwelling people with dementia experience significant unmet needs. However, family physician teams as primary health care gatekeepers for aging populations in China remain an underused resource for structured caregiver support.

**Objective:**

This hybrid effectiveness-implementation study aimed to evaluate a policy-aligned integration of the World Health Organization’s iSupport web-based program with China’s family physician contract services for informal dementia caregivers while systematically assessing implementation determinants using the Consolidated Framework for Implementation Research (CFIR).

**Methods:**

A cluster randomized controlled trial enrolled 120 informal caregivers of people with dementia in Beijing randomly assigned to either the intervention group (n=60) receiving the family physician team–guided iSupport intervention plus usual care or the control group (n=60) receiving usual care plus only iSupport registration. Caregiver burden (primary outcome) and quality of life, social support, and learning behaviors (secondary outcomes) were assessed at baseline and the postintervention time point. Intention-to-treat analysis was applied. The quantitative data were analyzed using linear mixed models and generalized linear mixed models. Implementation barriers and facilitators were explored through CFIR-guided thematic analysis of semistructured interviews with 34 stakeholders.

**Results:**

The intervention revealed no substantial between-group differences in caregiver burden (β=–3.80, 95% CI –11.25 to 3.65, *P*=.32), quality of life (β=–1.55, 95% CI –6.59 to 3.49, *P*=.55), or social support (β=0.85, 95% CI –2.86 to 4.56, *P*=.65). However, it demonstrated significantly improved learning behaviors in the intervention group (β=2.35, 95% CI 1.03-3.67; *P*<.001). CFIR analysis identified multilevel barriers: (1) policy-finance misalignment excluding dementia care from essential services, (2) digital adaptation gaps for older users, (3) lack of performance incentives in primary health care, (4) caregivers’ technological and time constraints, and (5) conflicts between standardized content and personalized needs.

**Conclusions:**

This iSupport-based primary health care intervention presents a policy-aligned support model for dementia caregivers in China. While core outcomes showed no significant improvement, enhanced learning behaviors suggest potential for caregiver empowerment. Key implementation insights focused on optimizing digital platforms, strengthening policy incentives, and personalizing support. These findings offer a scalable, policy-aligned model for dementia care management by leveraging primary health care networks, particularly in low- and middle-income countries.

**Trial Registration:**

Chinese Clinical Trial Registry ChiCTR2400084788; https://www.chictr.org.cn/showproj.html?proj=223091

## Introduction

### Background

Dementia, a progressive neurodegenerative disorder characterized by cognitive decline and behavioral disturbances, represents a growing global health challenge [1]. It affects approximately 55.2 million individuals worldwide in 2019, and cases may triple to 139 million by 2050 [2]. The associated economic burden is equally staggering, with global costs estimated at US $1.3 trillion in 2019 and potentially reaching US $2.8 trillion by 2030 [3]. This syndrome not only severely compromises patients’ activities of daily living but also imposes significant physical and psychological strain on caregivers, manifesting as increased rates of anxiety, depression, and chronic health conditions among this population [4,5]. Therefore, there is an urgent need for professional interventions to support dementia caregivers. The World Health Organization’s (WHO) *Global action plan on the public health response to dementia 2017-2025* identified caregiver support as a critical component of comprehensive dementia care [6].

In China, which is undergoing rapid population aging, dementia has emerged as the fifth leading cause of mortality [7]. Recent estimates indicate that 15.07 million Chinese adults aged >60 years are affected, with projected direct economic costs exceeding ¥2544.8 billion (approximately US $352 billion) by 2050 [7,8]. In response, China has implemented national policies to address dementia prevention and control, such as the Healthy China 2030 plan outline and the National Dementia Prevention and Treatment Program (2023-2025). In China, >90% of older adults live in home-community settings depending primarily on informal caregivers, underscoring the need for evidence-based, scalable support interventions through primary health care systems.

In 2019, the WHO launched iSupport for dementia [9], an evidence-based digital training program designed to mitigate caregiver burden through skill development and mental health support. This structured intervention comprises 5 core competency modules: Introduction to Dementia, Being a Carer, Caring for Me, Providing Everyday Care, and Dealing with Behavior Changes. Following its original English-language development, cross-cultural validation studies have demonstrated the program’s adaptability in diverse health care contexts, including in China [10], Portugal [11], India [12], the Netherlands [13], and Brazil [14]. Existing studies demonstrate mixed efficacy of the program in improving caregiver coping mechanisms and reducing psychological distress, with a notable lack of exploration of its integration within primary health care systems. Therefore, implementation science gaps persist regarding optimal delivery models in primary health care settings. Our study addresses this knowledge gap by integrating the validated Chinese iSupport adaptation within a family physician team–supervised primary health care framework.

Primary health care providers serve as the most accessible and cost-efficient frontline resource for informal dementia caregivers. China’s nationwide family physician contract service initiative, implemented in 2016, has established these professionals as core health gatekeepers in primary health care systems. Building on this policy foundation, we propose integrating iSupport into China’s family physician contract service to overcome 2 key adoption barriers: caregivers’ educational limitations and health literacy gaps. This hybrid implementation model combines digital education delivery with primary health care–based social support.

To our knowledge, the potential of primary health care providers to reduce dementia-related caregiving burdens in low- and middle-income countries remains understudied, creating a critical evidence gap given these countries’ accelerating dementia epidemic and strained health care systems.

### Objectives

This study had two objectives: (1) to assess the effectiveness of an iSupport-integrated primary health care intervention (aligned with family physician contract services) on caregiver burden, perceived social support, health-related quality of life, and learning behaviors; and (2) to identify key implementation determinants within the primary health care context for interventions aimed at informal caregivers of home-dwelling people with dementia in China. Using a theory-informed, mixed methods design that merges implementation science with pragmatic trial methodology, this study offers insights for scaling dementia caregiver interventions in primary health care settings.

## Methods

### Theoretical Framework

This study was grounded in social support theory, which collectively informed the research design and intervention framework. Social support theory emphasizes the critical role of emotional, informational, and tangible support provided by social networks in promoting individuals’ health and well-being [15,16]. The social support system consists of 3 main aspects: the social support subject, the social support object, and the social support mediator [17,18]. In this study, the subject of social support is the family physician team, which delivers the iSupport-based primary health care intervention. This intervention aims to equip caregivers with the knowledge and skills needed to improve their caregiving practices and self-care, ultimately reducing caregiving burden and enhancing their quality of life. The object of social support are the informal caregivers of people with dementia. Due to the overwhelming demands of caregiving, these individuals often experience deteriorating physical health and diminished self-care capacity, positioning them as a vulnerable group in need of societal support.

**Figure 1:**
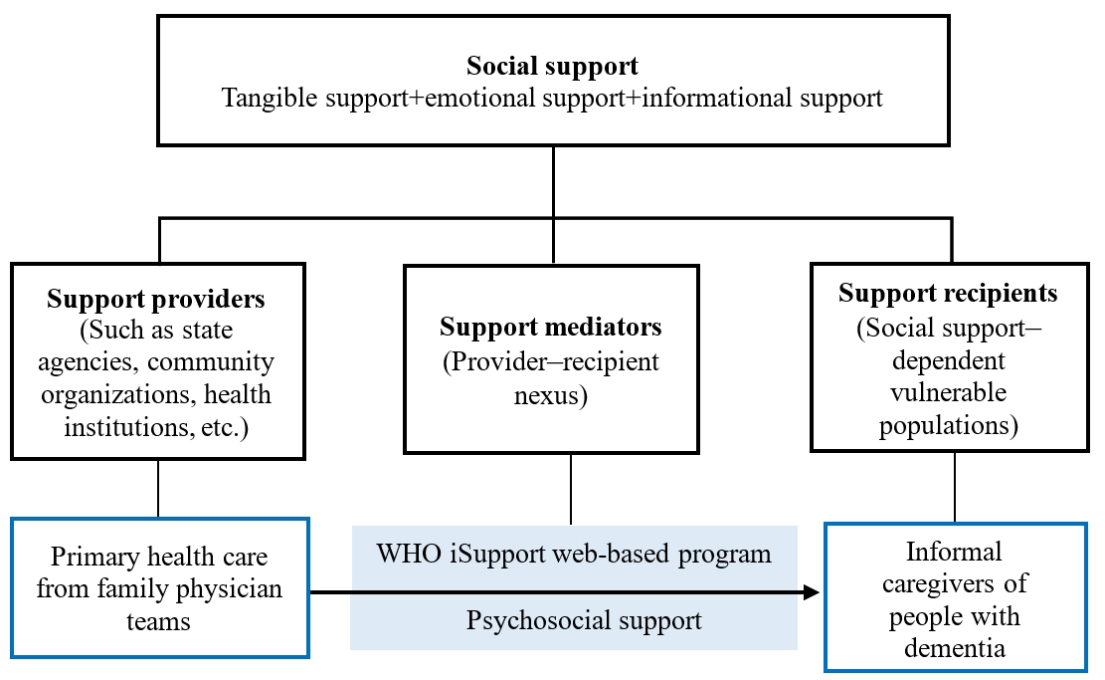
The theoretical framework in the study. WHO: World Health Organization.

The application of social support theories in this study is illustrated in Figure 1, which identifies 3 interconnected forms of support: tangible support, involving the provision of practical help; emotional support, achieved through communication and encouragement; and informational support from the iSupport program. The iSupport program, delivered through family physician contract services, serves as a mediator that enhances caregivers’ self-efficacy by providing targeted knowledge and skills. Simultaneously, the family physician team acts as a social support subject, offering emotional, informational, and tangible support to the caregiver, thereby addressing their multifaceted needs.

### Study Design

This study adopted a hybrid effectiveness-implementation research design [19], integrating a cluster randomized controlled trial and implementation research design, aiming to comprehensively evaluate the effectiveness of an iSupport-based primary health care intervention for informal dementia caregivers and the facilitators of and barriers to implementation. This study was implemented across urban communities and rural villages in Beijing for a 20-week intervention period.

### Design of the Cluster Randomized Controlled Trial (Stage 1)

#### Participants

Inclusion criteria were (1) primary caregivers for a home-dwelling individual with a confirmed dementia diagnosis, (2) unpaid caregiver status (spouse, adult child, relative, or friend), and (3) adequate digital literacy to use the intervention platforms (mobile phone or computer) and participate in study procedures. Exclusion criteria were (1) professional or volunteer caregivers; (2) the presence of physical, cognitive, or technological barriers preventing iSupport participation; and (3) unwillingness to participate.

Effect sizes reported in previous studies have mostly been >1 [20,21], prompting our use of a Cohen *d* value of 0.8. With an α of .05, power of 0.90, intraclass correlation coefficient of 0.05 [22], and 1:1 allocation, the required sample size was 48 per group. Accounting for a 20% attrition rate, the total target sample size was 116. We initially enrolled 120 caregivers of people with dementia via cluster randomization (n=60, 50% in the intervention group and n=60, 50% in the control group). During the trial, 15% (9/60) of the intervention group participants and 8% (5/60) of the control group participants withdrew, leaving 51 and 55 completers, respectively.

#### Participant Recruitment and Randomization

Community staff assisted with participant recruitment by providing access to the Civil Affairs Department’s disability registry and resident health records. Researchers then verified eligibility based on the inclusion and exclusion criteria. Eligible participants were sequentially grouped into clusters of 10 based on enrollment order, with each cluster assigned to a family physician team for health management. The study participants were recruited between May 2024 and July 2024.

Family physician teams served as key intervention implementers in China’s primary health care system. Given their direct caregiver engagement and service delivery role, we randomized at the team level (cluster) to ensure implementation consistency. Using a computer-generated random sequence, an independent researcher allocated teams to the intervention or control arms while maintaining allocation concealment. This approach preserved natural care delivery workflows while preventing selection bias, adhering to cluster randomized controlled trial guidelines.

#### Intervention

In China’s primary health care system, a standard family physician team consists of 3 core members: a general practitioner (family physician), a nurse, and a public health worker. These teams deliver essential public health services for older adults through community health centers or township health centers, including routine health examinations, health management programs, and chronic disease management (primarily targeting hypertension and diabetes). These services constitute the fundamental components of the conventional family physician contract service (usual care).

**Figure 2:**
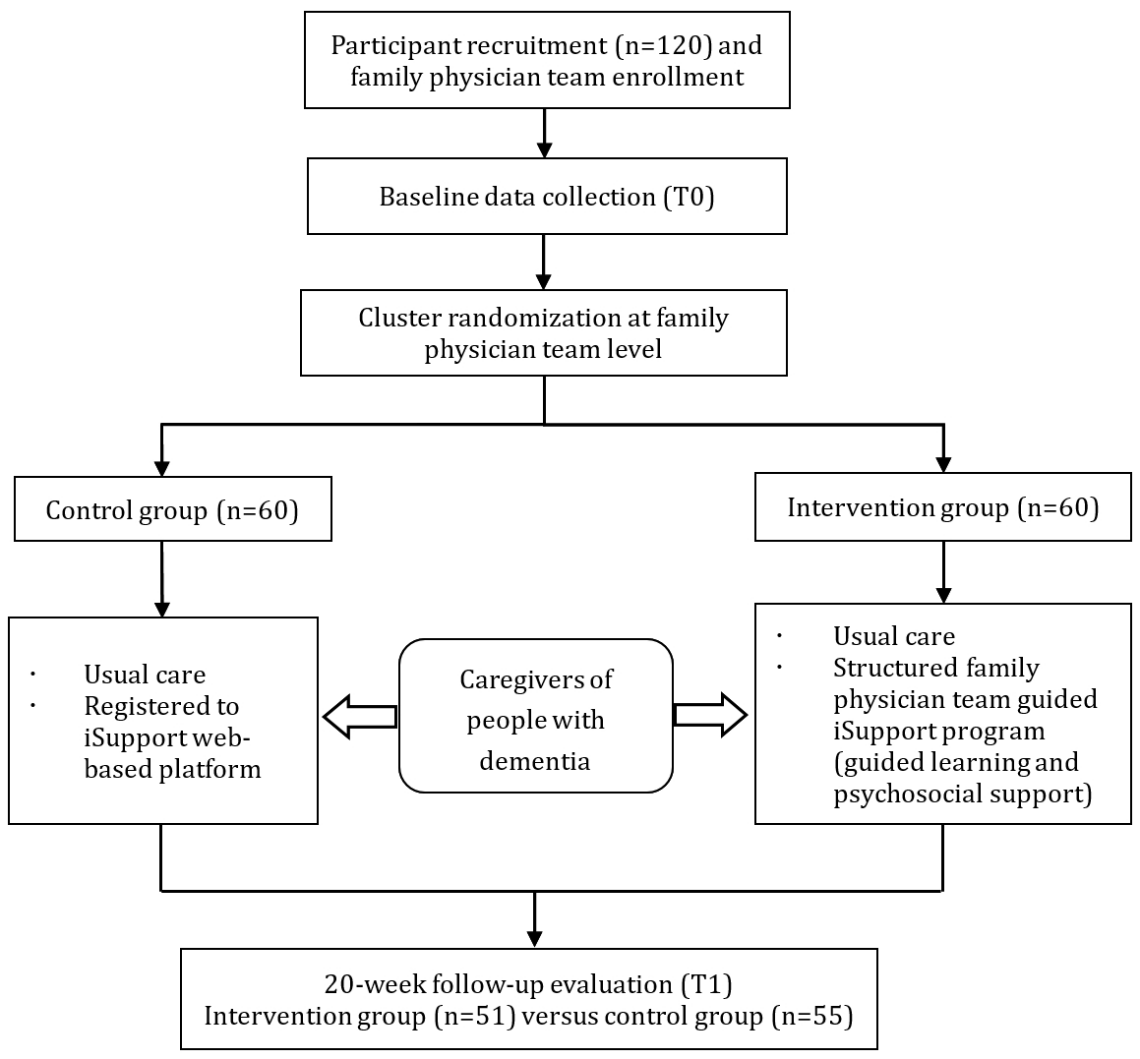
Flowchart of cluster randomized controlled trial.

In our intervention protocol, the intervention group received enhanced family physician contract services incorporating iSupport-based dementia caregiver support (iSupport-integrated primary health care intervention). The intervention consisted of two core components: (1) usual care, comprising standard public health services delivered by primary health care providers; and (2) a structured family physician team–guided iSupport program. The latter included (1) guided registration and systematic use of web-based educational modules and (2) psychosocial support through biweekly proactive follow-ups (conducted via WeChat or telephone). These follow-ups served multiple purposes: monitoring learning progress; providing personalized guidance on both didactic content and practical caregiving challenges; delivering systematic reminders to ensure timely module completion; and, ultimately, enhancing intervention adherence and motivation. To further optimize support, dedicated WeChat groups were established to facilitate immediate communication between caregivers and family physician teams, enabling real-time problem-solving and maintaining intervention fidelity throughout the program. Consistent with the 3- to 6-month intervention durations reported in previous studies and constrained by practical considerations, we implemented a 20-week intervention period. The intervention implementation process is presented in Figure 2.

In contrast, the control group received usual care only and was registered on the iSupport platform without any structured follow-ups, educational reinforcement, or active psychosocial support from family physician teams during the study period, thereby maintaining a natural observation condition throughout the study period.

#### Outcomes and Measures

##### Caregiving Burden (Primary Outcome)

The Caregiver Burden Inventory, a 24-item validated instrument, was used to assess caregiving burden of informal caregivers. As a well-validated and comprehensive measure, the Caregiver Burden Inventory has achieved international recognition in caregiver burden assessment [23]. The Chinese version of the scale has demonstrated excellent psychometric properties, including strong reliability and validity [24,25], confirming its appropriateness for use in this study.

##### Health-Related Quality of Life (Secondary Outcome)

We assessed health-related quality of life using the validated Chinese version of the brief version of the World Health Organization Quality of Life instrument [26]. It consists of 26 items. This measure has demonstrated excellent psychometric properties, with a Cronbach α of .874 indicating strong internal consistency. Domain-specific reliability was particularly robust, with Pearson correlation coefficients ranging from 0.89 (social relationships) to 0.95 (physical health) [27].

##### Social Support (Secondary Outcome)

We assessed social support using the validated 10-item Social Support Rating Scale by Xiao and Yang [28], which measures 3 dimensions: subjective, objective, and use of support. Total scores range from 12 to 66 (higher score=greater support). The Chinese version has shown excellent reliability, with internal consistency from 0.89 to 0.94 [29].

##### Learning Behaviors (Process Outcome)

Participant engagement was objectively measured through learning data automatically recorded by the iSupport platform.

#### Data Collection

Data were collected through questionnaires. A basic information questionnaire collected the demographic characteristics of study participants, such as age, gender, educational level, occupation, health status, and caregivers’ weekly caregiving hours. Validated scales were used to assess caregivers’ caregiving burden, health-related quality of life, and social support levels. Data collection was conducted at 2 time points: the baseline survey (collecting both basic information and outcome measures) and the final survey (focusing solely on outcome measures).

#### Data Analysis

Intention-to-treat analysis was applied. Parametric continuous data were analyzed using *t* tests with means and SDs, and nonparametric data were analyzed using medians and IQRs through Mann-Whitney *U* tests. Chi-square tests were used for categorical data (numbers and percentages). Two-tailed tests were used to report exact *P* values (except for *P*<.001). For normally distributed continuous outcomes (caregiver burden, health-related quality of life, and social support) with a hierarchical longitudinal structure, we used linear mixed-effects models. Learning behavior data (overdispersed count variables) were analyzed using negative binomial generalized linear mixed models, with negative binomial distribution and log-link function implemented. All models were adjusted for demographic and caregiving-related covariates, including sex, age, occupation, health status, and weekly caregiving hours.

### Implementation Determinant Analysis Using the Consolidated Framework for Implementation Research (Stage 2)

#### Overview

The second study phase used the Consolidated Framework for Implementation Research (CFIR) to systematically identify contextual factors influencing intervention implementation. This analysis examined 5 CFIR domains: innovation characteristics, outer setting, inner setting, individual characteristics, and implementation process [30]. Each domain includes specific subdomains (eg, innovation characteristics involve relative advantage and adaptability of the intervention, outer setting covers policies or regulations and sociocultural climate, inner setting includes organizational structure and resource accessibility, individual characteristics relate to implementers’ knowledge and motivation, and implementation process comprises planning and reflective adjustment).

#### Qualitative Data Collection

We used purposive sampling to conduct in-depth interviews with 34 key stakeholders involved in program implementation: 22 (65%) dementia caregivers (C01-C22), 6 (18%) family physicians (D01-D06), 2 (6%) health administrators (A01-A02) from the local health department, and 4 (12%) dementia care experts comprising 2 (50%) academic researchers in aging studies and 2 (50%) practicing geriatricians (E01-E04). To optimize participation, caregiver interviews (n=22, 65%) were conducted via telephone, whereas other participants (n=12, 35%) completed face-to-face interviews, accommodating preferences and logistical constraints.

Guided by the CFIR framework, we developed semistructured interview protocols through an iterative process combining literature review with preliminary quantitative findings [30,31]. Participants were particularly encouraged to discuss implementation barriers and facilitators, interprofessional communication patterns, time and resource requirements, and team coordination dynamics. After obtaining informed consent, all interviews were audio recorded and continued until reaching thematic saturation (achieved when 3 consecutive interviews yielded no new concepts).

#### Qualitative Analysis Methods

This study used a rigorous CFIR-informed thematic framework analysis comprising 2 phases. First, all audio recordings underwent professional verbatim transcription by trained research assistants while implementing strict anonymization protocols to remove all personal identifiers. Verbatim transcripts were systematically managed using NVivo (version 11.0; QSR International) to ensure analytical rigor throughout the coding process. Second, a comprehensive codebook was developed through iterative review of all CFIR constructs. The research team performed in-depth thematic synthesis by (1) conducting contextual re-examinations of coded excerpts within original interview transcripts, (2) categorizing emergent patterns as implementation facilitators or barriers through constant comparative analysis, and (3) producing finalized thematic frameworks accompanied by illustrative verbatim excerpts and analytical memos to ensure auditability.

### Ethical Considerations

This study was approved by the ethics committee of the Institute of Medical Information on November 28, 2023 (IMICAMS/05/23/HREC). The study strictly followed the World Medical Association Declaration of Helsinki and met international ethical standards for human subjects research. During recruitment, researchers distributed informed consent forms to participants to ensure that they were fully aware of the study. Caregivers who decide to participate were clearly informed of their rights by the researchers. Their participation in this study was voluntary, they had the right to leave at any time and without reason, and they were assured that this decision would not have any adverse effect on them or the person they cared for. They also had the right to refuse to answer questions if they felt uncomfortable. To protect privacy, researchers kept participants’ personal data and identity confidential: names and contact details were de-identified, sensitive data were password-protected, and no identifiable information was used in reports or publications without written consent. The study was registered with the Chinese Clinical Trial Registry (ChiCTR2400084788).

## Results

### Results of the Cluster Randomized Controlled Trial (Stage 1)

#### Baseline Characteristics of Trial Participants

As shown in Table 1, the intervention and control groups demonstrated balanced baseline characteristics across most measured variables (*P*>.05 in all cases), with the exception of caregiver age, where a statistically significant between-group difference was observed (*P*<.05). This finding suggests that randomization successfully achieved comparable groups in terms of all key demographic and clinical variables except for caregiver age, which was accounted for in subsequent adjusted analyses.

**Table 1:** Summary statistics of trial participants.

Characteristic			Intervention group (n=60)		Control group (n=60)		*P* value
**Sex, n (%)**							.19
	Male	20 (33)		27 (45)			
	Female	40 (67)		33 (55)			
Age (y), mean (SD)			57 (12.68)		52 (13.66)		.03
**Occupation, n (%)**							.51
	Farmer	19 (32)		17 (28)			
	Employment in nonpublic institutions	15 (25)		14 (23)			
	Employment in public institutions	14 (23)		9 (15)			
	Others	12 (20)		20 (33)			
**Health status, n (%)**							
	Hypertension	27 (45)		17 (28)		.09	
	Diabetes mellitus	11 (18)		10 (17)		.97	
	Hyperlipidemia	24 (40)		14 (23)		.11	
	Heart disease	13 (22)		5 (8)		.12	
	Cerebrovascular disease	5 (8)		1 (2)		.11	
**Weekly caregiving time (h), n (%)**							.35
	<10	12 (20)		8 (13)			
	10-20	9 (15)		8 (13)			
	20-30	2 (3)		7 (12)			
	30-40	1 (2)		4 (7)			
	40-50	6 (10)		5 (8)			
	≥50	30 (50)		28 (47)			
**Location, n (%)**							.19
	Rural area	33 (55)		40 (67)			
	Urban area	27 (45)		20 (33)			
**Educational level, n (%)**							.51
	Primary school or lower	3 (5)		10 (17)			
	Junior high school	21 (35)		23 (38)			
	Senior high school	12 (20)		11 (18)			
	College degree or higher	24 (40)		16 (27)			
**Marital status, n (%)**							.27
	Married or cohabiting	54 (90)		48 (80)			
	Single	2 (3)		5 (8)			
	Divorced	2 (3)		6 (10)			
	Widowed	2 (3)		1 (2)			
**Relationship with the patient, n (%)**							.23
	Spouse	12 (20)		10 (17)			
	Child	44 (73)		40 (67)			
	Friend	4 (7)		10 (17)			
**Outcome indicators, mean (SD)**							
	Caregiving burden (score of 0-96)	40.57 (19.39)		41.63 (19.41)		.76	
	Health-related quality of life (score of 24-120)	84.08 (13.72)		81.65 (13.47)		.33	
	Social support (score of 12-64)	35.85 (7.50)		37.63 (7.08)		.18	

#### Intervention Effects on Key Outcomes

Table 2 shows that the iSupport-integrated primary health care intervention did not yield statistically significant effects on either the primary outcome (caregiving burden) or secondary outcomes (health-related quality of life and social support) among informal dementia caregivers. Notably, the intervention did achieve a statistically significant improvement in caregivers’ learning behaviors during the 20-week implementation.

**Table 2:** Intervention effects on key outcomes.

Between-group effects by regression								Baseline, mean (SD)						20-week intervention period, mean (SD)			
Outcome and variable			β (95% CI)		*P* value		Intervention			Control			Intervention			Control	
**Caregiving burden**								40.57 (19.39)			41.63 (19.41)			37.08 (21.59)			41.95 (19.96)
	Group^a^	−0.58 (−7.40 to 6.24)		.88													
	Time^b^	0.32 (−4.95 to 5.58)		.91													
	Time × group	−3.80 (−11.25 to 3.65)		.32													
**Health-related quality of life**								84.08 (13.72)			81.65 (13.47)			82.30 (14.07)			81.42 (13.53)
	Group^a^	2.86 (−3.69 to 9.41)		.39													
	Time^b^	−0.23 (−3.79 to 3.33)		.90													
	Time × group	−1.55 (−6.59 to 3.49)		.55													
**Social support**								35.85 (7.50)			37.63 (7.08)			36.62 (7.84)			37.55 (7.98)
	Group^a^	−1.71 (−4.79 to 1.37)		.28													
	Time^b^	−0.08 (−2.71 to 2.54)		.95													
	Time × group	0.85 (−2.86 to 4.56)		.65													
**Learning behavior**								—^c^			—			23.15 (74.39)			3.90 (19.14)
	Group^a^	2.35 (1.03 to 3.67)		<.001													

^a^Indicated the between-group effect, adjusted for covariates including caregiver sex, age, occupation, health status, and weekly caregiving hours.

^b^Indicated the main effect of time, reflecting the changes in outcome indicators between the baseline survey and the 20-week post-intervention survey.

^c^Not applicable.

### Implementation Determinants Across CFIR Domains (Stage 2)

#### Innovation Characteristics Domain

Regarding the innovation relative advantage construct, caregivers recognized the intervention’s value primarily through its psychological support benefits, with one noting that “the main benefit was psychological support” (C08), whereas another acknowledged that “It did provide some knowledge I wouldn’t have learned otherwise” (C10). The web-based format offered accessibility advantages for time-constrained users as “I’m very busy—online courses are more convenient” (C19), although these benefits did not fully address core caregiving challenges. Many participants reported skepticism about the utility of the training (“Learning feels pointless”) and expressed a stronger demand for respite services rather than educational modules.

Regarding the innovation adaptability construct, significant implementation barriers emerged. Experts identified technological accessibility gaps for older caregivers, suggesting “more accessible formats like WeChat mini-programs or live streams” (E01). Cultural adaptation needs were apparent, with caregivers reporting content mismatches ranging from overly basic information as “After years of caregiving, I already know the basics” (C17) to limited applicability for complex cases as “My stubborn parent refuses most advice—many tips are useless” (C07). Practical utility concerns were raised, particularly the need for more disease-specific guidance, such as “I need experts to explain the disease itself” (C04).

The innovation complexity construct revealed cognitive barriers, particularly for low-literacy users, as one caregiver explained:

The content was too difficult—my education level isn’t high enough.

C21

The design quality and packaging construct included technical challenges such as page loading failures (“Pages wouldn’t load” [C22]) and progress tracking difficulties (“Finding where I left off was impossible” [C07]), along with content presentation concerns regarding text-heavy materials (“Slides with endless text make me more frustrated” [C08]) that prompted suggestions for alternative formats such as short videos from clinicians.

#### Outer Setting Domain

Stakeholder feedback from primary health care providers and dementia care experts primarily focused on the policies and financing constructs within this domain. Several national policies have recently emphasized dementia care, including the Healthy China 2030 plan (2016) and the National Dementia Prevention and Treatment Program (2023-2025), which specifically highlight the integration of dementia management into primary health care. However, unlike chronic disease programs, dementia care has not yet been incorporated into China’s essential public health service package or allocated dedicated funding, limiting its widespread implementation. The exclusion of dementia care metrics from primary health care performance evaluations has led to systemic deprioritization. This policy-practice misalignment, documented in implementation frameworks (eg, the CFIR), critically constrained real-world adoption.

Furthermore, ambiguous institutional accountability created coordination gaps, with one expert noting the lack of designated oversight:

There’s no designated department responsible for overseeing implementation.

E03

This lack of defined accountability mechanisms resulted in fragmented coordination and inadequate support for primary care providers attempting to operationalize the intervention. Moreover, practitioners faced challenges due to insufficient policy operationalization, expressing frustration with vague directives:

We need practical training systems and clear protocols, not just high-level mandates.

D01

The absence of specific, actionable guidelines left primary health care professionals without the necessary tools to effectively integrate dementia caregiver support into routine practice.

#### Inner Setting Domain

The inner setting analysis revealed a complex interplay of facilitators and barriers across 5 key constructs. Structural characteristics received partial community support, including practical assistance such as “80% reimbursement for diapers” (C09) and local problem-solving (“The village doctor helps resolve issues” [C17]), although significant gaps remained as some caregivers reported that “we’ve asked village doctors but received no help” (C01).

Implementation barriers included ineffective dissemination channels for older adults, with providers observing stagnant participation rates in health education activities (communications construct):

We held different types of health seminars for the elderly residents, but the same 30-40 people attend every event.

D03

Meanwhile, privacy barriers limited online support—such as “Can’t share sensitive issues publicly” (C01)—although informal networks offered emotional relief:

Chatting with family doctors and friends helps me vent.

C22

The relative priority construct was evident in the fact that dementia care’s nonmandated status affected clinical attention:

Without mandatory evaluation tasks, why prioritize non-essential work? I am so busy handling current workload.

D01

The analysis also identified critical gaps in incentive systems, with experts emphasizing the need “for economic/non-economic incentives AND training systems” (E01). Finally, knowledge accessibility challenges included caregivers’ requests for “instant advice for minor illnesses” (C22), contrasting with primary health care providers’ acknowledgment that “family doctors lack dementia-specific expertise” (D03). These findings collectively highlight multifaceted inner setting barriers requiring targeted implementation strategies.

#### Individual Characteristics Domain

Regarding perceived needs, caregiver attitudes varied substantially, ranging from self-reliant declarations such as “no external help needed” (C16) to requests for specialized training such as “teach us prevention strategies” (C19). These differences were further compounded by socioeconomic disparities, as noted by a health administrator:

[W]ealthier families hire aides; others use nursing homes.

HA01

Capability constraints emerged prominently, with age-related challenges affecting participation. Caregivers described difficulties with “forgetting to check the courses” (C14) and struggling with “small text” (C09), whereas family physicians acknowledged systemic knowledge gaps through statements such as “we lack dementia care expertise” (D03).

Opportunity barriers were equally significant, with caregivers facing severe time constraints (“no uninterrupted time to study” [C07]) and psychological strain manifesting as irritability (“I snap after repeating answers” [C03]) and depression (“nobody relieves my mental pressure” [C18]).

Finally, motivational drivers proved insufficient, with caregivers expressing disinterest in course content (“not interested” [C04 and C05]) and family physicians deprioritizing nonmandated tasks (“too busy for non-essential work” [D01]). These findings collectively demonstrate how individual-level factors create multiple layers of barriers to effective implementation.

#### Implementation Process Domain

All stakeholders universally emphasized the critical importance of supporting patients with dementia and their informal caregivers, recognizing this as essential for healthy aging initiatives. Primary health care providers and dementia experts further highlighted the implementation advantages of integrating the web-based iSupport program within routine family physician contract services: (1) convenience for users (“no need to visit community health centers”), (2) low operational burden (“minimal additional workload for family doctor teams”), and (3) little need for specialized support program training (reflecting and evaluating construct).

Contextual interference emerged in the fact that standardized online formats conflicted with personalized care needs, exemplified by a physician’s observation that “too many interfering factors complicate execution” (D01), underscoring the tension between scalability and individualization. Outcome uncertainties were highlighted by experts, cautioning that “behavior changes don’t guarantee reduced care burdens—long-term validation is needed” (E04), pointing to the complex relationship between knowledge acquisition and tangible caregiving improvements.

In addition, resistance dynamics from people with dementia created substantial implementation barriers, ranging from communication breakdowns (“Mom ignores all suggestions” [C07]) to outright rejection of support services (“he won’t accept hired help” [C09]). These process-related challenges collectively demonstrate the need for more adaptable implementation approaches that account for individual variability and longitudinal outcome measurement.

#### Summary

Table 3 presents the implementation determinants mapped across CFIR domains for the iSupport-integrated primary health care intervention.

**Table 3:** Summary of implementation determinants across Consolidated Framework for Implementation Research (CFIR) domains.

CFIR domain	Facilitators	Barriers	Mixed factors
Innovation characteristics	Psychological support benefits Online accessibility	Technological barriers for older users Content mismatches Cognitive complexity Technical design issues	Relative advantage recognized but limited impact
Outer setting	Designated as a priority in key national policy directives	Lack of performance metrics Unclear accountability Vague policy guidelines	—^a^
Inner setting	Partial community support and collaboration	Resource limitations Communication channel issues Competing priorities Incentive gaps	External caregiving support creates new stressors
Individual characteristics	—	Varied perceived needs Capability constraints Time poverty Low motivation	Socioeconomic disparities influence care
Implementation process	Convenience for users Low operational burden Little need for specialized training	Contextual interference Outcome uncertainties Patient resistance	Standardization versus customization tension

^a^Not applicable.

## Discussion

### Principal Findings

This study pioneered an innovative support model integrating the WHO iSupport program with China’s family physician contract service to address dementia caregiver needs in primary health care settings. While the intervention demonstrated significant efficacy in enhancing caregivers’ learning engagement, it yielded no measurable improvements in caregiving burden, quality of life, or social support outcomes. CFIR analysis identified multilevel barriers: (1) policy-finance misalignment excluding dementia care from essential services, (2) digital adaptation gaps for older users, (3) lack of performance incentives in primary health care, (4) caregivers’ technological and time constraints, and (5) conflicts between standardized content and personalized needs, highlighting implementation challenges in supporting informal dementia caregivers through the primary health care system.

First, the intervention’s success in enhancing learning engagement stemmed from family physicians’ dual capacity as health care coordinators and trusted community partners [32,33]. Their biweekly follow-ups, rooted in established clinical relationships and community health networks, effectively countered self-directed learning limitations through structured reminders that reduced forgetfulness, particularly among older caregivers. Social support theory explains this mechanism—the combination of tangible support (eg, reminders and progress tracking) and emotional support (eg, consistent communication) enhanced adherence while reducing caregiver isolation [34,35]. Based on the CFIR analysis, key moderating factors were identified to enhance learning engagement among older caregivers. The results indicated that simplified formats, such as voice guided modules and short videos, were essential to address technological and cognitive barriers in this population. This necessitates adaptive implementation strategies, including high-visuality interfaces for older users, enhanced offline support for technology-challenged caregivers, and literacy-tiered support systems [36]. These findings highlight the central tension in digital health interventions between scalability through technological standardization and effectiveness through personalized adaptation.

Second, this study substantiates the inherent limitations of stand-alone digital interventions for dementia caregivers, demonstrating that purely online approaches yield suboptimal outcomes without integrated interpersonal support and cultural adaptation, as evidenced by the UK iSupport trial’s minimal engagement (mean 4 log-ins in 6 months) and null clinical effects [37]. Facilitated models exemplified by nurse-guided peer support achieved significant mental health improvements, highlighting the critical need to address digital literacy disparities and psychosocial isolation among older caregivers [10].

While our intervention’s biweekly physician follow-ups partially mitigated self-directed learning barriers, systemic limitations remained. Critically, the iSupport program’s time-intensive design, particularly its reliance on slide-based and audio formats, proved fatiguing for older caregivers while yielding limited perceived benefits. Many participants viewed the program as burdensome, with qualitative feedback indicating a preference for respite services over educational content. These challenges highlight fundamental gaps that cannot be resolved through e-learning alone, necessitating policy-level interventions (such as mandated integration of caregiver support into primary care services) or structural support (such as the development of community-based respite care networks).

Third, while our study observed significant improvements in caregivers’ learning behaviors throughout the intervention period, the psychoeducational components and coping strategy training within the WHO iSupport program showed limited measurable impact on core caregiver outcomes, including caregiving burden, quality of life, and perceived social support. While the 20-week intervention aligns with international trials [12,37], there is emerging evidence showing delayed anxiety reduction [11,38], suggesting that such timelines inadequately capture psychological outcomes and learned skills need longer practice and adaptation to translate into tangible well-being outcomes. In addition, high-intensity caregivers exhibited diminished intervention engagement due to chronic fatigue, a phenomenon amplified by short-term frameworks’ inability to sustain behavior changes against persistent stressors.

Furthermore, despite being assigned to the control group, some highly motivated participants may have independently accessed and learned from the iSupport web-based platform without the structured reminders and support from family physician teams. Conversely, certain participants in the intervention group may have remained disengaged despite receiving systematic psychosocial support. This phenomenon was corroborated by the qualitative findings, which revealed a misalignment between caregivers’ immediate practical needs and the structured content of the iSupport modules. These observations underscore the importance of identifying priority subgroups with high perceived needs when implementing targeted interventions using standardized web-based resources such as iSupport.

Finally, we recognize that, although the outcomes chosen in our study match those of earlier dementia caregiver intervention studies [4,5,10,13], they may not fully capture the subtle effects of a learning-focused intervention. For example, the chosen health-related outcomes may be less sensitive to short-term changes in learning behaviors; the iSupport web-based intervention focused mainly on knowledge delivery with limited guidance on applying practical skills. This may indicate a measurement-system misfit as burden stems from complex systemic factors beyond education, quality of life depends on environmental changes, and newly learned strategies require extended practice before showing measurable effects. Therefore, dementia caregiver support may require concurrent environmental and policy-level modifications.

### Strengths and Limitations

This study used a hybrid effectiveness-implementation design to evaluate a novel integration of the WHO’s iSupport program within China’s family physician contract service, simultaneously assessing clinical outcomes and implementation determinants. The intervention established a policy-aligned, evidence-based model for informal dementia caregiver support in primary health care, demonstrating how global guidelines can be adapted to local health care infrastructures. By combining rigorous outcome evaluation with real-world implementation science, this study provides a model for transnational intervention adaptation in China’s primary health care setting while identifying key policy and contextual barriers.

However, this study has 3 key limitations that warrant caution and may inform future implementations of similar web-based psychoeducation interventions for caregivers of people with dementia. First, while the web-based delivery model improved accessibility without substantially increasing primary health care providers’ workload, its pragmatic reliance on online delivery limited hands-on skill coaching and emotional support, potentially reducing the practical application of acquired knowledge in real caregiving situations. Variability in primary health care providers’ dementia expertise and supervisory approaches further contributed to inconsistent implementation fidelity. Second, differential engagement with the iSupport modules may have introduced dose-response variability that diluted intervention effects. Third, the digital literacy requirement in participant selection likely introduced selection bias by excluding caregivers with limited technological proficiency, thereby restricting the findings to more digitally adept populations.

### Policy Implications and Future Research Recommendations

This study proposes 3 key intervention strategies to address dementia caregiver support challenges in real-world implementation. The first strategy involves policy-level integration to motivate family physician teams in providing proactive caregiver health management. This can be achieved by incorporating dementia care into existing public health programs, establishing performance-based incentives, and developing health–social service coordination mechanisms. The second strategy involves adapting the iSupport platform for China’s home-based care context by developing localized digital solutions such as WeChat mini programs with voice-guided interfaces and microlearning content (3- to 5-minute videos). These would be complemented by offline resource distribution through primary health care centers and tiered support modules to accommodate caregivers’ diverse needs and digital skills. The third strategy emphasizes an integrated support network linking primary health care, peer support, and mental health services. The model implements standardized screenings, organized peer interventions, and formalized community collaborations to create a sustainable, multidimensional support system for dementia caregivers.

Three recommendations are proposed for future research on dementia caregiver interventions. First, intervention design should primarily address the critical balance between digital scalability and dementia care’s relational aspects, with hybrid digital-human support models showing the greatest promise. Second, while extended study durations may be necessary to detect meaningful health outcome changes from web-based educational interventions, researchers must carefully calibrate observation periods to maintain participant engagement and prevent attrition. Third, outcome measures should be more precisely aligned with intervention content, particularly through domain-specific assessments of caregiver skill acquisition and care recipient functional improvements.

### Conclusions

This study demonstrates both the potential and challenges of integrating the WHO’s iSupport program into China’s family physician system to support informal dementia caregivers. While failing to immediately improve traditional outcomes (caregiver burden, quality of life, and social support), it significantly enhanced learning engagement, suggesting value in caregiver empowerment potential. The findings reveal critical gaps between global guidelines and local realities, particularly in digital platforms’ design, personalized intervention components, support networks, and outcome measurement. For low- and middle-income countries, we recommend leveraging primary health care networks to deliver tiered support while developing metrics that capture both competency gains and long-term impacts.

## Appendix

Multimedia Appendix 1 CONSORT-eHEALTH checklist (V 1.6.1).

## Abbreviations

CFIR: Consolidated Framework for Implementation Research
WHO: World Health Organization

## Data Availability

The datasets generated or analyzed during this study are available from the corresponding author on reasonable request.
