# Supplementary material for: Supporting Informal Dementia Caregivers Through an iSupport Web-Based Primary Health Care Intervention: Hybrid Effectiveness-Implementation Mixed Methods Study
Source: J Med Internet Res. 2025 Sep 9;27:e77688. doi: 10.2196/77688 (PMC12457867; doi:10.2196/77688)
Supplement: Multimedia Appendix 1 [file jmir_v27i1e77688_app1.pdf]

CONSORT-EHEALTH (V 1.6.1) - Submission/Publication Form

The CONSORT-EHEALTH checklist is intended for authors of randomized trials evaluating web-based and Internet-based applications/interventions, including mobile interventions, electronic games (incl multiplayer games), social media, certain telehealth applications, and other interactive and/or networked electronic applications. Some of the items (e.g. all subitems under item 5 - description of the intervention) may also be applicable for other study designs.

The goal of the CONSORT EHEALTH checklist and guideline is to be
a) a guide for reporting for authors of RCTs,
b) to form a basis for appraisal of an ehealth trial (in terms of validity)

CONSORT-EHEALTH items/subitems are MANDATORY reporting items for studies published in the Journal of Medical Internet Research and other journals / scientific societies endorsing the checklist.

Items numbered 1., 2., 3., 4a., 4b etc are original CONSORT or CONSORT-NPT (non-pharmacologic treatment) items.
Items with Roman numerals (i., ii, iii, iv etc.) are CONSORT-EHEALTH extensions/clarifications.

As the CONSORT-EHEALTH checklist is still considered in a formative stage, we would ask that you also RATE ON A SCALE OF 1-5 how important/useful you feel each item is FOR THE PURPOSE OF THE CHECKLIST and reporting guideline (optional).

Mandatory reporting items are marked with a red *.
In the textboxes, either copy & paste the relevant sections from your manuscript into this form - please include any quotes from your manuscript in QUOTATION MARKS,
or answer directly by providing additional information not in the manuscript, or elaborating on why the item was not relevant for this study.

YOUR ANSWERS WILL BE PUBLISHED AS A SUPPLEMENTARY FILE TO YOUR PUBLICATION IN JMIR AND ARE CONSIDERED PART OF YOUR PUBLICATION (IF ACCEPTED).
Please fill in these questions diligently. Information will not be copyedited, so please use proper spelling and grammar, use correct capitalization, and avoid abbreviations.

DO NOT FORGET TO SAVE AS PDF _AND_ CLICK THE SUBMIT BUTTON SO YOUR ANSWERS ARE IN OUR DATABASE !!!

Citation Suggestion (if you append the pdf as Appendix we suggest to cite this paper in the caption):
Eysenbach G, CONSORT-EHEALTH Group
CONSORT-EHEALTH: Improving and Standardizing Evaluation Reports of Web-based and Mobile Health Interventions
J Med Internet Res 2011;13(4):e126

URL: http://www.jmir.org/2011/4/e126/
doi: 10.2196/jmir.1923
PMID: 22209829

登录 Google 即可保存进度。了解详情

* 表示必填

Your name *

First Last

Shasha Yuan

Primary Affiliation (short), City, Country *

University of Toronto, Toronto, Canada

Chinese Academy of Medical Sciences & Pekir

Your e-mail address *

abc@gmail.com

yuanshasha417@163.com

Title of your manuscript *

Provide the (draft) title of your manuscript.

Supporting informal dementia caregivers in China: Lessons from a hybrid effectiveness–implementation trial of an iSupport web–based primary healthcare intervention

Name of your App/Software/Intervention *

If there is a short and a long/alternate name, write the short name first and add the long name in brackets.

WHO (World Health Organization) iSupport we

Evaluated Version (if any)

e.g. "V1", "Release 2017-03-01", "Version 2.0.27913"

您的回答

Language(s) *

What language is the intervention/app in? If multiple languages are available, separate by comma (e.g. "English, French")

Chinese, English

URL of your Intervention Website or App

e.g. a direct link to the mobile app on app in appstore (itunes, Google Play), or URL of the website. If the intervention is a DVD or hardware, you can also link to an Amazon page.

http://www.cncdec.cn/

URL of an image/screenshot (optional)

您的回答

Accessibility *

Can an enduser access the intervention presently?

- (selected) access is free and open
- access only for special usergroups, not open
- access is open to everyone, but requires payment/subscription/in-app purchases
- app/intervention no longer accessible
- 其他:

Primary Medical Indication/Disease/Condition *

e.g. "Stress", "Diabetes", or define the target group in brackets after the condition, e.g. "Autism (Parents of children with)", "Alzheimers (Informal Caregivers of)"

Dementia (Informal Caregivers of)

Primary Outcomes measured in trial *

comma-separated list of primary outcomes reported in the trial

Caregiver burden

Secondary/other outcomes

Are there any other outcomes the intervention is expected to affect?

quality of life, social support, and learning behaviors (secondary outcomes)

Recommended "Dose" *

What do the instructions for users say on how often the app should be used?

- ○ Approximately Daily
- ○ Approximately Weekly
- ○ Approximately Monthly
- ○ Approximately Yearly
- ◉ "as needed"
- ○ 其他:

Approx. Percentage of Users (starters) still using the app as recommended after 3 months *

- ○ unknown / not evaluated
- ○ 0-10%
- ○ 11-20%
- ○ 21-30%
- ○ 31-40%
- ○ 41-50%
- ◉ 51-60%
- ○ 61-70%
- ○ 71%-80%
- ○ 81-90%
- ○ 91-100%
- ○ 其他:

Overall, was the app/intervention effective? *

- ○ yes: all primary outcomes were significantly better in intervention group vs control
- ○ partly: SOME primary outcomes were significantly better in intervention group vs control
- ◉ no statistically significant difference between control and intervention
- ○ potentially harmful: control was significantly better than intervention in one or more outcomes
- ○ inconclusive: more research is needed
- ○ 其他:

Article Preparation Status/Stage *

At which stage in your article preparation are you currently (at the time you fill in this form)

- ○ not submitted yet - in early draft status
- ◉ not submitted yet - in late draft status, just before submission
- ○ submitted to a journal but not reviewed yet
- ○ submitted to a journal and after receiving initial reviewer comments
- ○ submitted to a journal and accepted, but not published yet
- ○ published
- ○ 其他:

Journal *

If you already know where you will submit this paper (or if it is already submitted), please provide the journal name (if it is not JMIR, provide the journal name under "other")

- ○ not submitted yet / unclear where I will submit this
- ◉ Journal of Medical Internet Research (JMIR)
- ○ JMIR mHealth and UHealth
- ○ JMIR Serious Games
- ○ JMIR Mental Health
- ○ JMIR Public Health
- ○ JMIR Formative Research
- ○ Other JMIR sister journal
- ○ 其他:

Is this a full powered effectiveness trial or a pilot/feasibility trial? *

- ○ Pilot/feasibility
- ◉ Fully powered

Manuscript tracking number *

If this is a JMIR submission, please provide the manuscript tracking number under "other" (The ms tracking number can be found in the submission acknowledgement email, or when you login as author in JMIR. If the paper is already published in JMIR, then the ms tracking number is the four-digit number at the end of the DOI, to be found at the bottom of each published article in JMIR)

- ◉ no ms number (yet) / not (yet) submitted to / published in JMIR
- ○ 其他:

TITLE AND ABSTRACT

1a) TITLE: Identification as a randomized trial in the title

1a) Does your paper address CONSORT item 1a? *

I.e does the title contain the phrase "Randomized Controlled Trial"? (if not, explain the reason under "other")

- (x) yes
- ( ) 其他:

1a-i) Identify the mode of delivery in the title

Identify the mode of delivery. Preferably use “web-based” and/or “mobile” and/or “electronic game” in the title. Avoid ambiguous terms like “online”, “virtual”, “interactive”. Use “Internet-based” only if Intervention includes non-web-based Internet components (e.g. email), use “computer-based” or “electronic” only if offline products are used. Use “virtual” only in the context of “virtual reality” (3-D worlds). Use “online” only in the context of “online support groups”. Complement or substitute product names with broader terms for the class of products (such as “mobile” or “smart phone” instead of “iphone”), especially if the application runs on different platforms.

	1	2	3	4	5	
subitem not at all important	( )	( )	( )	(x)	( )	essential

清除所选内容

Does your paper address subitem 1a-i? *

Copy and paste relevant sections from manuscript title (include quotes in quotation marks "like this" to indicate direct quotes from your manuscript), or elaborate on this item by providing additional information not in the ms, or briefly explain why the item is not applicable/relevant for your study

"Supporting informal dementia caregivers in China: Lessons from a hybrid effectiveness–implementation trial of an iSupport web–based primary healthcare intervention"

1a-ii) Non-web-based components or important co-interventions in title

Mention non-web-based components or important co-interventions in title, if any (e.g., “with telephone support”).

	1	2	3	4	5	
subitem not at all important	○	○	○	◉	○	essential

清除所选内容

Does your paper address subitem 1a-ii?

Copy and paste relevant sections from manuscript title (include quotes in quotation marks "like this" to indicate direct quotes from your manuscript), or elaborate on this item by providing additional information not in the ms, or briefly explain why the item is not applicable/relevant for your study

"an iSupport web–based primary healthcare intervention"

1a-iii) Primary condition or target group in the title

Mention primary condition or target group in the title, if any (e.g., “for children with Type I Diabetes”) Example: A Web-based and Mobile Intervention with Telephone Support for Children with Type I Diabetes: Randomized Controlled Trial

	1	2	3	4	5	
subitem not at all important	○	○	○	○	◉	essential

清除所选内容

Does your paper address subitem 1a-iii? *

Copy and paste relevant sections from manuscript title (include quotes in quotation marks "like this" to indicate direct quotes from your manuscript), or elaborate on this item by providing additional information not in the ms, or briefly explain why the item is not applicable/relevant for your study

"informal dementia caregivers"

1b) ABSTRACT: Structured summary of trial design, methods, results, and conclusions

NPT extension: Description of experimental treatment, comparator, care providers, centers, and blinding status.

1b-i) Key features/functionalities/components of the intervention and comparator in the METHODS section of the ABSTRACT

Mention key features/functionalities/components of the intervention and comparator in the abstract. If possible, also mention theories and principles used for designing the site. Keep in mind the needs of systematic reviewers and indexers by including important synonyms. (Note: Only report in the abstract what the main paper is reporting. If this information is missing from the main body of text, consider adding it)

	1	2	3	4	5	
subitem not at all important	○	○	○	○	◉	essential

清除所选内容

Does your paper address subitem 1b-i? *

Copy and paste relevant sections from the manuscript abstract (include quotes in quotation marks "like this" to indicate direct quotes from your manuscript), or elaborate on this item by providing additional information not in the ms, or briefly explain why the item is not applicable/relevant for your study

"A cluster randomized controlled trial enrolled 120 informal caregivers of PWD in Beijing, randomly assigned to either the intervention group (family doctor contract services: 20–week iSupport training, enhanced health management, and usual care; n=60) or the control group (usual care only; n=60). Caregiver burden (primary outcome), quality of life, social support, and learning behaviors (secondary outcomes) were assessed at baseline and post–intervention."

1b-ii) Level of human involvement in the METHODS section of the ABSTRACT

Clarify the level of human involvement in the abstract, e.g., use phrases like “fully automated” vs. “therapist/nurse/care provider/physician-assisted” (mention number and expertise of providers involved, if any). (Note: Only report in the abstract what the main paper is reporting. If this information is missing from the main body of text, consider adding it)

	1	2	3	4	5	
subitem not at all important	○	○	○	◉	○	essential

清除所选内容

Does your paper address subitem 1b-ii?

Copy and paste relevant sections from the manuscript abstract (include quotes in quotation marks "like this" to indicate direct quotes from your manuscript), or elaborate on this item by providing additional information not in the ms, or briefly explain why the item is not applicable/relevant for your study

"A cluster randomized controlled trial enrolled 120 informal caregivers of PWD in Beijing, randomly assigned to either the intervention group (family doctor contract services: 20–week iSupport training, enhanced health management, and usual care; n=60) or the control group (usual care only; n=60). Caregiver burden (primary outcome), quality of life, social support, and learning behaviors (secondary outcomes) were assessed at baseline and post–intervention."

1b-iii) Open vs. closed, web-based (self-assessment) vs. face-to-face assessments in the METHODS section of the ABSTRACT

Mention how participants were recruited (online vs. offline), e.g., from an open access website or from a clinic or a closed online user group (closed usergroup trial), and clarify if this was a purely web-based trial, or there were face-to-face components (as part of the intervention or for assessment). Clearly say if outcomes were self-assessed through questionnaires (as common in web-based trials). Note: In traditional offline trials, an open trial (open-label trial) is a type of clinical trial in which both the researchers and participants know which treatment is being administered. To avoid confusion, use “blinded” or “unblinded” to indicated the level of blinding instead of “open”, as “open” in web-based trials usually refers to “open access” (i.e. participants can self-enrol). (Note: Only report in the abstract what the main paper is reporting. If this information is missing from the main body of text, consider adding it)

	1	2	3	4	5	
subitem not at all important	○	○	●	○	○	essential

清除所选内容

Does your paper address subitem 1b-iii?

Copy and paste relevant sections from the manuscript abstract (include quotes in quotation marks "like this" to indicate direct quotes from your manuscript), or elaborate on this item by providing additional information not in the ms, or briefly explain why the item is not applicable/relevant for your study

"A cluster randomized controlled trial enrolled 120 informal caregivers of PWD in Beijing, randomly assigned to either the intervention group (family doctor contract services: 20–week iSupport training, enhanced health management, and usual care; n=60) or the control group (usual care only; n=60). Caregiver burden (primary outcome), quality of life, social support, and learning behaviors (secondary outcomes) were assessed at baseline and post–intervention."

1b-iv) RESULTS section in abstract must contain use data

Report number of participants enrolled/assessed in each group, the use/uptake of the intervention (e.g., attrition/adherence metrics, use over time, number of logins etc.), in addition to primary/secondary outcomes. (Note: Only report in the abstract what the main paper is reporting. If this information is missing from the main body of text, consider adding it)

	1	2	3	4	5	
subitem not at all important	○	○	○	◉	○	essential

清除所选内容

Does your paper address subitem 1b-iv?

Copy and paste relevant sections from the manuscript abstract (include quotes in quotation marks "like this" to indicate direct quotes from your manuscript), or elaborate on this item by providing additional information not in the ms, or briefly explain why the item is not applicable/relevant for your study

"The intervention revealed no significant between–group differences in caregiver burden (β=–0.58, 95%CI:–7.40, 6.24), quality of life (β=–0.23, 95%CI: −3.79, 3.33), or social support (β=–0.08, 95%CI:–2.71, 2.54). But it demonstrated significantly improved learning behaviors in the intervention group (β=2.35, 95%CI: 1.03, 3.67, P<.001). CFIR analysis identified multi–level barriers: (1) policy–finance misalignment excluding dementia care from essential services; (2) digital adaptation gaps for older users; (3) lack of performance incentives in primary healthcare; (4) caregivers' technological/time constraints; and (5) conflicts between standardized content and personalized needs." We reported the number of participants enrolled/assessed in each group in the Methods section. It is more better than reported in the Results section.

1b-v) CONCLUSIONS/DISCUSSION in abstract for negative trials

Conclusions/Discussions in abstract for negative trials: Discuss the primary outcome - if the trial is negative (primary outcome not changed), and the intervention was not used, discuss whether negative results are attributable to lack of uptake and discuss reasons. (Note: Only report in the abstract what the main paper is reporting. If this information is missing from the main body of text, consider adding it)

	1	2	3	4	5	
subitem not at all important	○	○	○	◉	○	essential

清除所选内容

Does your paper address subitem 1b-v?

Copy and paste relevant sections from the manuscript abstract (include quotes in quotation marks "like this" to indicate direct quotes from your manuscript), or elaborate on this item by providing additional information not in the ms, or briefly explain why the item is not applicable/relevant for your study

"This iSupport–based primary healthcare intervention presents a policy–aligned support model for dementia caregivers in China. While core outcomes showed no significant improvement, enhanced learning behaviors suggest potential for caregiver empowerment. Key implementation insights reveal optimizing digital platforms, strengthening policy incentives, and personalizing support. These findings offer a scalable, policy–aligned model for dementia care management by leveraging primary healthcare networks, particularly in LMICs."

INTRODUCTION

2a) In INTRODUCTION: Scientific background and explanation of rationale

2a-i) Problem and the type of system/solution

Describe the problem and the type of system/solution that is object of the study: intended as stand-alone intervention vs. incorporated in broader health care program? Intended for a particular patient population? Goals of the intervention, e.g., being more cost-effective to other interventions, replace or complement other solutions? (Note: Details about the intervention are provided in “Methods” under 5)

	1	2	3	4	5	
subitem not at all important	○	○	○	◉	○	essential

清除所选内容

Does your paper address subitem 2a-i? *

Copy and paste relevant sections from the manuscript (include quotes in quotation marks "like this" to indicate direct quotes from your manuscript), or elaborate on this item by providing additional information not in the ms, or briefly explain why the item is not applicable/relevant for your study

"Dementia, a progressive neurodegenerative disorder characterized by cognitive decline and behavioral disturbances, represents a growing global health challenge[1]. Affecting approximately 55.2 million individuals worldwide in 2019, cases may triple to 139 million by 2050[2]. The associated economic burden is equally staggering, with global costs estimated at $1.3 trillion globally in 2019, and potentially reaching $2.8 trillion by 2030[3]. This syndrome not only severely compromises patients' activities of daily living but also imposes significant physical and psychological strain on caregivers, manifesting as increased rates of anxiety, depression, and chronic health conditions among this population[4,5]. Therefore, there is an urgent need for professional interventions to support dementia caregivers. The World Health Organization (WHO) Global Action Plan on the Public Health Response to Dementia 2017–2025 identified caregiver support as a critical component of comprehensive dementia care[6].

In China, where population aging is occurring at an unprecedented rate, dementia has emerged as the fifth leading cause of mortality[7]. Recent estimates indicate 15.07 million Chinese adults aged over 60 years are affected, with projected direct economic costs exceeding 2544.8 billion yuan by 2050[7,8]. In response, China has implemented national policies to address dementia prevention and control such as "Healthy China 2030" Plan Outline and the National Dementia Prevention and Treatment Program (2023–2025). In China, over 90% of older adults live in home–community settings, depending primarily on informal caregivers, underscoring evidence–based, scalable support interventions through primary healthcare systems.

In 2019, WHO launched iSupport for Dementia[9], an evidence–based digital training program designed to mitigate caregiver burden through skill development and mental health support. This structured intervention comprises five core competency modules: "Introduction to Dementia, Being a Carer, Caring for Me, Providing Everyday Care, and Dealing with Behavior Changes". Following its original English development, cross–cultural validation studies have demonstrated the program's adaptability in diverse healthcare contexts, including China[10], Portugal[11], India[12], the Netherlands[13], Brazil[14], etc. Existing studies demonstrate mixed efficacy of the program in improving caregiver coping mechanisms and reducing psychological distress, with a notable lack of exploration regarding its integration within primary healthcare systems. Therefore, implementation science gaps persist regarding optimal delivery models in primary healthcare settings. Our study addresses this knowledge gap by integrating the validated Chinese iSupport adaptation within a family doctor teams–supervised primary healthcare framework.

Primary healthcare providers serve as the most accessible and cost–efficient frontline resource for informal dementia caregivers. China's nationwide family doctor contract service initiative, implemented since 2016, has established these professionals as core health gatekeepers in primary healthcare systems. Building on this policy foundation, we propose integrating iSupport into China's family doctor contract service to overcome two key adoption barriers: caregivers' educational limitations and health literacy gaps. This hybrid implementation model combines digital education delivery with primary healthcare–based social support.

To our knowledge, the potential of primary healthcare providers to reduce dementia–related caregiving burdens in LMICs remains understudied, creating a critical evidence gap given the region's accelerating dementia epidemic and strained healthcare systems.

Objectives
This study has dual objectives: (1) to assess the effectiveness of an iSupport–integrated

[illegible]
primary healthcare intervention (aligned with family doctor contract service) on caregiver burden, perceived social support, health–related quality of life and learning behavior; and (2) to identify key implementation determinants within primary healthcare context for informal caregivers of home–dwelling PWD in China. Employing a theory–informed, mixed–methods design that merges implementation science with pragmatic trial methodology, this study offers insights for scaling dementia caregiver interventions in primary healthcare settings."

2a-ii) Scientific background, rationale: What is known about the (type of) system

Scientific background, rationale: What is known about the (type of) system that is the object of the study (be sure to discuss the use of similar systems for other conditions/diagnoses, if appropiate), motivation for the study, i.e. what are the reasons for and what is the context for this specific study, from which stakeholder viewpoint is the study performed, potential impact of findings [2]. Briefly justify the choice of the comparator.

	1	2	3	4	5	
subitem not at all important	○	○	○	◉	○	essential

清除所选内容

Does your paper address subitem 2a-ii? *

Copy and paste relevant sections from the manuscript (include quotes in quotation marks "like this" to indicate direct quotes from your manuscript), or elaborate on this item by providing additional information not in the ms, or briefly explain why the item is not applicable/relevant for your study

"In 2019, WHO launched iSupport for Dementia[9], an evidence–based digital training program designed to mitigate caregiver burden through skill development and mental health support. This structured intervention comprises five core competency modules: "Introduction to Dementia, Being a Carer, Caring for Me, Providing Everyday Care, and Dealing with Behavior Changes". Following its original English development, cross–cultural validation studies have demonstrated the program's adaptability in diverse healthcare contexts, including China[10], Portugal[11], India[12], the Netherlands[13], Brazil[14], etc. Existing studies demonstrate mixed efficacy of the program in improving caregiver coping mechanisms and reducing psychological distress, with a notable lack of exploration regarding its integration within primary healthcare systems. Therefore, implementation science gaps persist regarding optimal delivery models in primary healthcare settings. Our study addresses this knowledge gap by integrating the validated Chinese iSupport adaptation within a family doctor teams–supervised primary healthcare framework.

Primary healthcare providers serve as the most accessible and cost–efficient frontline resource for informal dementia caregivers. China's nationwide family doctor contract service initiative, implemented since 2016, has established these professionals as core health gatekeepers in primary healthcare systems. Building on this policy foundation, we propose integrating iSupport into China's family doctor contract service to overcome two key adoption barriers: caregivers' educational limitations and health literacy gaps. This hybrid implementation model combines digital education delivery with primary healthcare–based social support.

To our knowledge, the potential of primary healthcare providers to reduce dementia–related caregiving burdens in LMICs remains understudied, creating a critical evidence gap given the region's accelerating dementia epidemic and strained healthcare systems."

2b) In INTRODUCTION: Specific objectives or hypotheses

Does your paper address CONSORT subitem 2b? *

Copy and paste relevant sections from the manuscript (include quotes in quotation marks "like this" to indicate direct quotes from your manuscript), or elaborate on this item by providing additional information not in the ms, or briefly explain why the item is not applicable/relevant for your study

"This study has dual objectives: (1) to assess the effectiveness of an iSupport–integrated primary healthcare intervention (aligned with family doctor contract service) on caregiver burden, perceived social support, health–related quality of life and learning behavior; and (2) to identify key implementation determinants within primary healthcare context for informal caregivers of home–dwelling PWD in China. Employing a theory–informed, mixed–methods design that merges implementation science with pragmatic trial methodology, this study offers insights for scaling dementia caregiver interventions in primary healthcare settings."

METHODS

3a) Description of trial design (such as parallel, factorial) including allocation ratio

Does your paper address CONSORT subitem 3a? *

Copy and paste relevant sections from the manuscript (include quotes in quotation marks "like this" to indicate direct quotes from your manuscript), or elaborate on this item by providing additional information not in the ms, or briefly explain why the item is not applicable/relevant for your study

"Study design
This study adopts a hybrid effectiveness–implementation research design[19], integrating a cluster randomized controlled trial and implementation research design, aiming to comprehensively evaluate the effectiveness of an iSupport–based primary healthcare intervention for informal dementia caregivers and the facilitators and barriers of implementation. The study was implemented across urban communities and rural villages in Beijing, with a 20–week intervention period."

3b) Important changes to methods after trial commencement (such as eligibility criteria), with reasons

Does your paper address CONSORT subitem 3b? *

Copy and paste relevant sections from the manuscript (include quotes in quotation marks "like this" to indicate direct quotes from your manuscript), or elaborate on this item by providing additional information not in the ms, or briefly explain why the item is not applicable/relevant for your study

NA. There were no important changes to methods after trial commencement in this study.

3b-i) Bug fixes, Downtimes, Content Changes

Bug fixes, Downtimes, Content Changes: ehealth systems are often dynamic systems. A description of changes to methods therefore also includes important changes made on the intervention or comparator during the trial (e.g., major bug fixes or changes in the functionality or content) (5-iii) and other "unexpected events" that may have influenced study design such as staff changes, system failures/downtimes, etc. [2].

	1	2	3	4	5	
subitem not at all important	○	○	○	○	○	essential

Does your paper address subitem 3b-i?

Copy and paste relevant sections from the manuscript (include quotes in quotation marks "like this" to indicate direct quotes from your manuscript), or elaborate on this item by providing additional information not in the ms, or briefly explain why the item is not applicable/relevant for your study

NA. Participants could directly access the web-page to conduct self-learning. The web-based iSupport program is relatively stable.

4a) Eligibility criteria for participants

Does your paper address CONSORT subitem 4a? *

Copy and paste relevant sections from the manuscript (include quotes in quotation marks "like this" to indicate direct quotes from your manuscript), or elaborate on this item by providing additional information not in the ms, or briefly explain why the item is not applicable/relevant for your study

"Participants
Inclusion criteria: (1) Primary caregiver for a home–dwelling individual with confirmed dementia diagnosis; (2) Unpaid caregiver status (spouse, adult child, relative, or friend); (3) Adequate digital literacy to use intervention platforms (mobile/computer) and participate in study procedures. Exclusion criteria: (1) Professional or volunteer caregivers; (3) Physical, cognitive, or technological barriers preventing iSupport participation; (3) Unwillingness to participate."

4a-i) Computer / Internet literacy

Computer / Internet literacy is often an implicit "de facto" eligibility criterion - this should be explicitly clarified.

	1	2	3	4	5	
subitem not at all important	○	○	●	○	○	essential

清除所选内容

Does your paper address subitem 4a-i?

Copy and paste relevant sections from the manuscript (include quotes in quotation marks "like this" to indicate direct quotes from your manuscript), or elaborate on this item by providing additional information not in the ms, or briefly explain why the item is not applicable/relevant for your study

In this study, we included participants " (3) Adequate digital literacy to use intervention platforms (mobile/computer) and participate in study procedures."

4a-ii) Open vs. closed, web-based vs. face-to-face assessments:

Open vs. closed, web-based vs. face-to-face assessments: Mention how participants were recruited (online vs. offline), e.g., from an open access website or from a clinic, and clarify if this was a purely web-based trial, or there were face-to-face components (as part of the intervention or for assessment), i.e., to what degree got the study team to know the participant. In online-only trials, clarify if participants were quasi-anonymous and whether having multiple identities was possible or whether technical or logistical measures (e.g., cookies, email confirmation, phone calls) were used to detect/prevent these.

	1	2	3	4	5	
subitem not at all important	○	○	○	◉	○	essential

清除所选内容

Does your paper address subitem 4a-ii? *

Copy and paste relevant sections from the manuscript (include quotes in quotation marks "like this" to indicate direct quotes from your manuscript), or elaborate on this item by providing additional information not in the ms, or briefly explain why the item is not applicable/relevant for your study

"Community staff assisted with participant recruitment by providing access to the Civil Affairs Department's disability registry and resident health records. Researchers then verified eligibility based on the inclusion/exclusion criteria. Eligible participants were sequentially assigned in groups of 10 based on enrollment order, with each cluster assigned to a family doctor team for health management. The study participants were recruited between May and July 2024."

4a-iii) Information giving during recruitment

Information given during recruitment. Specify how participants were briefed for recruitment and in the informed consent procedures (e.g., publish the informed consent documentation as appendix, see also item X26), as this information may have an effect on user self-selection, user expectation and may also bias results.

	1	2	3	4	5	
subitem not at all important	○	○	◉	○	○	essential

清除所选内容

Does your paper address subitem 4a-iii?

Copy and paste relevant sections from the manuscript (include quotes in quotation marks "like this" to indicate direct quotes from your manuscript), or elaborate on this item by providing additional information not in the ms, or briefly explain why the item is not applicable/relevant for your study

During recruitment, researchers distributed informed consent forms to participants to ensure that they are fully aware of the study. Caregivers who decide to participate were clearly informed of their rights by the researchers. Their participation in this study was voluntary and they have the right to leave at any time and without reason, and that this decision did not have any adverse effect on them or the person they care for. They also have the right to refuse to answer questions if they feel uncomfortable.

4b) Settings and locations where the data were collected

Does your paper address CONSORT subitem 4b? *

Copy and paste relevant sections from the manuscript (include quotes in quotation marks "like this" to indicate direct quotes from your manuscript), or elaborate on this item by providing additional information not in the ms, or briefly explain why the item is not applicable/relevant for your study

"The study was implemented across urban communities and rural villages in Beijing, with a 20–week intervention period."

4b-i) Report if outcomes were (self-)assessed through online questionnaires

Clearly report if outcomes were (self-)assessed through online questionnaires (as common in web-based trials) or otherwise.

	1	2	3	4	5	
subitem not at all important	○	○	○	◉	○	essential

清除所选内容

Does your paper address subitem 4b-i? *

Copy and paste relevant sections from the manuscript (include quotes in quotation marks "like this" to indicate direct quotes from your manuscript), or elaborate on this item by providing additional information not in the ms, or briefly explain why the item is not applicable/relevant for your study

"Data collection
Data were collected through questionnaire surveys. A basic information questionnaire collects demographic characteristics of study participants, such as age, gender, education level, occupation, chronic disease status, caregivers' weekly caregiving hours, etc. Validated scales were used to assess caregivers' caregiving burden, health–related quality of life, and social support levels. Data collection was conducted at two time points: the baseline survey (collecting both basic information and outcome measures) and the final survey (focusing solely on outcome measures)."

4b-ii) Report how institutional affiliations are displayed

Report how institutional affiliations are displayed to potential participants [on ehealth media], as affiliations with prestigious hospitals or universities may affect volunteer rates, use, and reactions with regards to an intervention.(Not a required item – describe only if this may bias results)

	1	2	3	4	5	
subitem not at all important	○	◉	○	○	○	essential

清除所选内容

Does your paper address subitem 4b-ii?

Copy and paste relevant sections from the manuscript (include quotes in quotation marks "like this" to indicate direct quotes from your manuscript), or elaborate on this item by providing additional information not in the ms, or briefly explain why the item is not applicable/relevant for your study

您的回答

5) The interventions for each group with sufficient details to allow replication, including how and when they were actually administered

5-i) Mention names, credential, affiliations of the developers, sponsors, and owners

Mention names, credential, affiliations of the developers, sponsors, and owners [6] (if authors/evaluators are owners or developer of the software, this needs to be declared in a “Conflict of interest” section or mentioned elsewhere in the manuscript).

	1	2	3	4	5	
subitem not at all important	○	○	○	◉	○	essential

清除所选内容

Does your paper address subitem 5-i?

Copy and paste relevant sections from the manuscript (include quotes in quotation marks "like this" to indicate direct quotes from your manuscript), or elaborate on this item by providing additional information not in the ms, or briefly explain why the item is not applicable/relevant for your study

"Intervention
In China’s primary healthcare system, a standard family doctor team consists of three core members: a general practitioner (family doctor), a nurse, and a public health worker. These teams deliver essential public health services for older adults through community health centers or township health centers, including routine health examinations, health management programs, and chronic disease management (primarily targeting hypertension and diabetes). These services constitute the fundamental components of conventional family doctor contract service (usual care).

In our intervention protocol, the intervention group received enhanced family doctor contract service incorporating iSupport–based dementia caregiver support. This augmented care model included all standard services plus proactive biweekly follow–ups conducted through WeChat or telephone, with three specific objectives: (1) ensuring timely completion of iSupport educational modules through structured reminders; (2) monitoring learning progression while addressing both course–related questions and practical caregiving difficulties; and (3) delivering psychosocial support to enhance engagement, including professional guidance for course content and caregiving challenges. To optimize communication, dedicated WeChat groups connected caregivers with their family doctor teams, enabling immediate problem–solving throughout the intervention. The control group continued receiving usual care without proactive engagement. Their family doctor teams maintained a responsive (rather than proactive) approach, addressing caregiver inquiries only when specifically requested, thereby preserving naturalistic care delivery conditions. Consistent with the 3–6 month intervention durations reported in prior studies and constrained by practical considerations, we implemented a 20–week intervention period. The intervention implementation process is presented in Figure 2." The other information regarding iSupport was presented in the Background part.

5-ii) Describe the history/development process

Describe the history/development process of the application and previous formative evaluations (e.g., focus groups, usability testing), as these will have an impact on adoption/use rates and help with interpreting results.

	1	2	3	4	5	
subitem not at all important	○	○	○	◉	○	essential

清除所选内容

Does your paper address subitem 5-ii?

Copy and paste relevant sections from the manuscript (include quotes in quotation marks "like this" to indicate direct quotes from your manuscript), or elaborate on this item by providing additional information not in the ms, or briefly explain why the item is not applicable/relevant for your study

您的回答

5-iii) Revisions and updating

Revisions and updating. Clearly mention the date and/or version number of the application/intervention (and comparator, if applicable) evaluated, or describe whether the intervention underwent major changes during the evaluation process, or whether the development and/or content was "frozen" during the trial. Describe dynamic components such as news feeds or changing content which may have an impact on the replicability of the intervention (for unexpected events see item 3b).

	1	2	3	4	5	
subitem not at all important	○	○	○	○	○	essential

Does your paper address subitem 5-iii?

Copy and paste relevant sections from the manuscript (include quotes in quotation marks "like this" to indicate direct quotes from your manuscript), or elaborate on this item by providing additional information not in the ms, or briefly explain why the item is not applicable/relevant for your study

"Intervention
In China's primary healthcare system, a standard family doctor team consists of three core members: a general practitioner (family doctor), a nurse, and a public health worker. These teams deliver essential public health services for older adults through community health centers or township health centers, including routine health examinations, health management programs, and chronic disease management (primarily targeting hypertension and diabetes). These services constitute the fundamental components of conventional family doctor contract service (usual care).

In our intervention protocol, the intervention group received enhanced family doctor contract service incorporating iSupport–based dementia caregiver support. This augmented care model included all standard services plus proactive biweekly follow–ups conducted through WeChat or telephone, with three specific objectives: (1) ensuring timely completion of iSupport educational modules through structured reminders; (2) monitoring learning progression while addressing both course–related questions and practical caregiving difficulties; and (3) delivering psychosocial support to enhance engagement, including professional guidance for course content and caregiving challenges. To optimize communication, dedicated WeChat groups connected caregivers with their family doctor teams, enabling immediate problem–solving throughout the intervention. The control group continued receiving usual care without proactive engagement. Their family doctor teams maintained a responsive (rather than proactive) approach, addressing caregiver inquiries only when specifically requested, thereby preserving naturalistic care delivery conditions. Consistent with the 3–6 month intervention durations reported in prior studies and constrained by practical considerations, we implemented a 20–week intervention period. The intervention implementation process is presented in Figure 2."

5-iv) Quality assurance methods

Provide information on quality assurance methods to ensure accuracy and quality of information provided [1], if applicable.

	1	2	3	4	5	
subitem not at all important	○	○	○	◉	○	essential

清除所选内容

Does your paper address subitem 5-iv?

Copy and paste relevant sections from the manuscript (include quotes in quotation marks "like this" to indicate direct quotes from your manuscript), or elaborate on this item by providing additional information not in the ms, or briefly explain why the item is not applicable/relevant for your study

We combined the role of family doctor teams in the iSupport web-based intervention as described in the "Intervention"

5-v) Ensure replicability by publishing the source code, and/or providing screenshots/screen-capture video, and/or providing flowcharts of the algorithms used

Ensure replicability by publishing the source code, and/or providing screenshots/screen-capture video, and/or providing flowcharts of the algorithms used. Replicability (i.e., other researchers should in principle be able to replicate the study) is a hallmark of scientific reporting.

	1	2	3	4	5	
subitem not at all important	○	○	○	●	○	essential

清除所选内容

Does your paper address subitem 5-v?

Copy and paste relevant sections from the manuscript (include quotes in quotation marks "like this" to indicate direct quotes from your manuscript), or elaborate on this item by providing additional information not in the ms, or briefly explain why the item is not applicable/relevant for your study

您的回答

5-vi) Digital preservation

Digital preservation: Provide the URL of the application, but as the intervention is likely to change or disappear over the course of the years; also make sure the intervention is archived (Internet Archive, webcitation.org, and/or publishing the source code or screenshots/videos alongside the article). As pages behind login screens cannot be archived, consider creating demo pages which are accessible without login.

	1	2	3	4	5	
subitem not at all important	○	○	○	◉	○	essential

清除所选内容

Does your paper address subitem 5-vi?

Copy and paste relevant sections from the manuscript (include quotes in quotation marks "like this" to indicate direct quotes from your manuscript), or elaborate on this item by providing additional information not in the ms, or briefly explain why the item is not applicable/relevant for your study

您的回答

5-vii) Access

Access: Describe how participants accessed the application, in what setting/context, if they had to pay (or were paid) or not, whether they had to be a member of specific group. If known, describe how participants obtained “access to the platform and Internet” [1]. To ensure access for editors/reviewers/readers, consider to provide a “backdoor” login account or demo mode for reviewers/readers to explore the application (also important for archiving purposes, see vi).

	1	2	3	4	5	
subitem not at all important	○	○	○	◉	○	essential

清除所选内容

Does your paper address subitem 5-vii? *

Copy and paste relevant sections from the manuscript (include quotes in quotation marks "like this" to indicate direct quotes from your manuscript), or elaborate on this item by providing additional information not in the ms, or briefly explain why the item is not applicable/relevant for your study

The researchers help the participants registered in the iSupport web-based program, then they could access to the web page as needed.

5-viii) Mode of delivery, features/functionalities/components of the intervention and comparator, and the theoretical framework

Describe mode of delivery, features/functionalities/components of the intervention and comparator, and the theoretical framework [6] used to design them (instructional strategy [1], behaviour change techniques, persuasive features, etc., see e.g., [7, 8] for terminology). This includes an in-depth description of the content (including where it is coming from and who developed it) [1],” whether [and how] it is tailored to individual circumstances and allows users to track their progress and receive feedback” [6]. This also includes a description of communication delivery channels and – if computer-mediated communication is a component – whether communication was synchronous or asynchronous [6]. It also includes information on presentation strategies [1], including page design principles, average amount of text on pages, presence of hyperlinks to other resources, etc. [1].

	1	2	3	4	5	
subitem not at all important	○	○	○	◉	○	essential

清除所选内容

Does your paper address subitem 5-viii? *

Copy and paste relevant sections from the manuscript (include quotes in quotation marks "like this" to indicate direct quotes from your manuscript), or elaborate on this item by providing additional information not in the ms, or briefly explain why the item is not applicable/relevant for your study

"Theoretical framework
This study is grounded in social support theory, which collectively inform the research design and intervention framework. Social support theory emphasizes the critical role of emotional, informational, and tangible support provided by social networks in promoting individuals' health and well–being[15,16]. The social support system consists of three main aspects: the social support subject, the social support object and the social support mediator[17,18]. In this study, the subject of social support is the family doctor team, which delivers the iSupport–based primary healthcare intervention and enhanced health management care. This intervention aims to equip caregivers with the knowledge and skills needed to improve their caregiving practices and self–care, ultimately reducing caregiving burden and enhancing their quality of life. The object of social support is the informal caregivers of PWD. Due to the overwhelming demands of caregiving, these individuals often experience deteriorating physical health and diminished self–care capacity, positioning them as a vulnerable group in need of societal support.
The application of social support theories in this study is illustrated in Figure 1. The iSupport program, delivered through family doctor contract services, serves as a mediator that enhances caregivers' self–efficacy by providing targeted knowledge and skills. Simultaneously, the family doctor team acts as a social support subject, offering emotional, informational, and practical assistance to caregivers, thereby addressing their multifaceted needs."

"In our intervention protocol, the intervention group received enhanced family doctor contract service incorporating iSupport–based dementia caregiver support. This augmented care model included all standard services plus proactive biweekly follow–ups conducted through WeChat or telephone, with three specific objectives: (1) ensuring timely completion of iSupport educational modules through structured reminders; (2) monitoring learning progression while addressing both course–related questions and practical caregiving difficulties; and (3) delivering psychosocial support to enhance engagement, including professional guidance for course content and caregiving challenges. To optimize communication, dedicated WeChat groups connected caregivers with their family doctor teams, enabling immediate problem–solving throughout the intervention. The control group continued receiving usual care without proactive engagement. Their family doctor teams maintained a responsive (rather than proactive) approach, addressing caregiver inquiries only when specifically requested, thereby preserving naturalistic care delivery conditions. Consistent with the 3–6 month intervention durations reported in prior studies and constrained by practical considerations, we implemented a 20–week intervention period. The intervention implementation process is presented in Figure 2."

5-ix) Describe use parameters

Describe use parameters (e.g., intended “doses” and optimal timing for use). Clarify what instructions or recommendations were given to the user, e.g., regarding timing, frequency, heaviness of use, if any, or was the intervention used ad libitum.

	1	2	3	4	5	
subitem not at all important	○	○	○	○	○	essential

Does your paper address subitem 5-ix?

Copy and paste relevant sections from the manuscript (include quotes in quotation marks "like this" to indicate direct quotes from your manuscript), or elaborate on this item by providing additional information not in the ms, or briefly explain why the item is not applicable/relevant for your study

您的回答

5-x) Clarify the level of human involvement

Clarify the level of human involvement (care providers or health professionals, also technical assistance) in the e-intervention or as co-intervention (detail number and expertise of professionals involved, if any, as well as “type of assistance offered, the timing and frequency of the support, how it is initiated, and the medium by which the assistance is delivered”. It may be necessary to distinguish between the level of human involvement required for the trial, and the level of human involvement required for a routine application outside of a RCT setting (discuss under item 21 – generalizability).

	1	2	3	4	5	
subitem not at all important	○	○	○	○	○	essential

Does your paper address subitem 5-x?

Copy and paste relevant sections from the manuscript (include quotes in quotation marks "like this" to indicate direct quotes from your manuscript), or elaborate on this item by providing additional information not in the ms, or briefly explain why the item is not applicable/relevant for your study

"In our intervention protocol, the intervention group received enhanced family doctor contract service incorporating iSupport–based dementia caregiver support. This augmented care model included all standard services plus proactive biweekly follow–ups conducted through WeChat or telephone, with three specific objectives: (1) ensuring timely completion of iSupport educational modules through structured reminders; (2) monitoring learning progression while addressing both course–related questions and practical caregiving difficulties; and (3) delivering psychosocial support to enhance engagement, including professional guidance for course content and caregiving challenges. To optimize communication, dedicated WeChat groups connected caregivers with their family doctor teams, enabling immediate problem–solving throughout the intervention. The control group continued receiving usual care without proactive engagement. Their family doctor teams maintained a responsive (rather than proactive) approach, addressing caregiver inquiries only when specifically requested, thereby preserving naturalistic care delivery conditions. Consistent with the 3–6 month intervention durations reported in prior studies and constrained by practical considerations, we implemented a 20–week intervention period. The intervention implementation process is presented in Figure 2."

5-xi) Report any prompts/reminders used

Report any prompts/reminders used: Clarify if there were prompts (letters, emails, phone calls, SMS) to use the application, what triggered them, frequency etc. It may be necessary to distinguish between the level of prompts/reminders required for the trial, and the level of prompts/reminders for a routine application outside of a RCT setting (discuss under item 21 – generalizability).

	1	2	3	4	5	
subitem not at all important	○	○	◉	○	○	essential

清除所选内容

Does your paper address subitem 5-xi? *

Copy and paste relevant sections from the manuscript (include quotes in quotation marks "like this" to indicate direct quotes from your manuscript), or elaborate on this item by providing additional information not in the ms, or briefly explain why the item is not applicable/relevant for your study

"In our intervention protocol, the intervention group received enhanced family doctor contract service incorporating iSupport–based dementia caregiver support. This augmented care model included all standard services plus proactive biweekly follow–ups conducted through WeChat or telephone, with three specific objectives: (1) ensuring timely completion of iSupport educational modules through structured reminders; (2) monitoring learning progression while addressing both course–related questions and practical caregiving difficulties; and (3) delivering psychosocial support to enhance engagement, including professional guidance for course content and caregiving challenges. To optimize communication, dedicated WeChat groups connected caregivers with their family doctor teams, enabling immediate problem–solving throughout the intervention. The control group continued receiving usual care without proactive engagement. Their family doctor teams maintained a responsive (rather than proactive) approach, addressing caregiver inquiries only when specifically requested, thereby preserving naturalistic care delivery conditions. Consistent with the 3–6 month intervention durations reported in prior studies and constrained by practical considerations, we implemented a 20–week intervention period. The intervention implementation process is presented in Figure 2."

5-xii) Describe any co-interventions (incl. training/support)

Describe any co-interventions (incl. training/support): Clearly state any interventions that are provided in addition to the targeted eHealth intervention, as ehealth intervention may not be designed as stand-alone intervention. This includes training sessions and support [1]. It may be necessary to distinguish between the level of training required for the trial, and the level of training for a routine application outside of a RCT setting (discuss under item 21 – generalizability.

	1	2	3	4	5	
subitem not at all important	○	○	○	◉	○	essential

清除所选内容

Does your paper address subitem 5-xii? *

Copy and paste relevant sections from the manuscript (include quotes in quotation marks "like this" to indicate direct quotes from your manuscript), or elaborate on this item by providing additional information not in the ms, or briefly explain why the item is not applicable/relevant for your study

"In our intervention protocol, the intervention group received enhanced family doctor contract service incorporating iSupport–based dementia caregiver support. This augmented care model included all standard services plus proactive biweekly follow–ups conducted through WeChat or telephone, with three specific objectives: (1) ensuring timely completion of iSupport educational modules through structured reminders; (2) monitoring learning progression while addressing both course–related questions and practical caregiving difficulties; and (3) delivering psychosocial support to enhance engagement, including professional guidance for course content and caregiving challenges. To optimize communication, dedicated WeChat groups connected caregivers with their family doctor teams, enabling immediate problem–solving throughout the intervention. The control group continued receiving usual care without proactive engagement. Their family doctor teams maintained a responsive (rather than proactive) approach, addressing caregiver inquiries only when specifically requested, thereby preserving naturalistic care delivery conditions. Consistent with the 3–6 month intervention durations reported in prior studies and constrained by practical considerations, we implemented a 20–week intervention period. The intervention implementation process is presented in Figure 2."

6a) Completely defined pre-specified primary and secondary outcome measures, including how and when they were assessed

Does your paper address CONSORT subitem 6a? *

Copy and paste relevant sections from the manuscript (include quotes in quotation marks "like this" to indicate direct quotes from your manuscript), or elaborate on this item by providing additional information not in the ms, or briefly explain why the item is not applicable/relevant for your study

"Outcomes and measures
(1) Caregiving burden (primary outcome): The Caregiver Burden Inventory (CBI) was used to assess caregiving burden of informal caregivers. As a well–validated and comprehensive measure, the CBI has achieved international recognition in caregiver burden assessment[23]. The Chinese version of the scale has demonstrated excellent psychometric properties, including strong reliability and validity[24,25], confirming its appropriateness for use in this study.

(2) Health–related quality of life (secondary outcome): We assessed health–related quality of life using the validated Chinese version of the WHOQOL–BREF instrument[26]. This measure demonstrates excellent psychometric properties, with a Cronbach's α of 0.874 indicating strong internal consistency. Domain–specific reliability was particularly robust with pearson correlation coefficients ranging from 0.89 (social relationships) to 0.95 (physical health) [27].

(3) Social support (secondary outcome): We assessed social support using Xiao's validated 10–item Social Support Rating Scale [28], which measures three dimensions: subjective, objective, and utilization of support. Total scores range from 12–66 (higher=greater support). The Chinese version shows excellent reliability with internal consistency from 0.89 to 0.94[29].

(4) Learning behavior (process outcome): Participant engagement was objectively measured through learning time data automatically recorded by the iSupport platform."

6a-i) Online questionnaires: describe if they were validated for online use and apply CHERRIES items to describe how the questionnaires were designed/deployed

If outcomes were obtained through online questionnaires, describe if they were validated for online use and apply CHERRIES items to describe how the questionnaires were designed/deployed [9].

	1	2	3	4	5	
subitem not at all important	○	○	◉	○	○	essential

清除所选内容

Does your paper address subitem 6a-i?

Copy and paste relevant sections from manuscript text

您的回答

6a-ii) Describe whether and how “use” (including intensity of use/dosage) was defined/measured/monitored

Describe whether and how “use” (including intensity of use/dosage) was defined/measured/monitored (logins, logfile analysis, etc.). Use/adoption metrics are important process outcomes that should be reported in any ehealth trial.

	1	2	3	4	5	
subitem not at all important	○	○	◉	○	○	essential

清除所选内容

Does your paper address subitem 6a-ii?

Copy and paste relevant sections from manuscript text

Self learning and "In our intervention protocol, the intervention group received enhanced family doctor contract service incorporating iSupport–based dementia caregiver support. This augmented care model included all standard services plus proactive biweekly follow–ups conducted through WeChat or telephone"

6a-iii) Describe whether, how, and when qualitative feedback from participants was obtained

Describe whether, how, and when qualitative feedback from participants was obtained (e.g., through emails, feedback forms, interviews, focus groups).

	1	2	3	4	5	
subitem not at all important	○	○	○	◉	○	essential

清除所选内容

Does your paper address subitem 6a-iii?

Copy and paste relevant sections from manuscript text

"Implementation Determinants Analysis Using CFIR Framework (Stage II)
The second study phase employed the CFIR to systematically identify contextual factors influencing intervention implementation. This analysis examined five CFIR domains: innovation characteristics, outer setting, inner setting, individual characteristics, and implementation process[30]."

6b) Any changes to trial outcomes after the trial commenced, with reasons

Does your paper address CONSORT subitem 6b? *

Copy and paste relevant sections from the manuscript (include quotes in quotation marks "like this" to indicate direct quotes from your manuscript), or elaborate on this item by providing additional information not in the ms, or briefly explain why the item is not applicable/relevant for your study

NA. There was no changes after trial commenced.

7a) How sample size was determined

NPT: When applicable, details of whether and how the clustering by care provides or centers was addressed

7a-i) Describe whether and how expected attrition was taken into account when calculating the sample size

Describe whether and how expected attrition was taken into account when calculating the sample size.

	1	2	3	4	5	
subitem not at all important	○	○	○	○	◉	essential

清除所选内容

Does your paper address subitem 7a-i?

Copy and paste relevant sections from manuscript title (include quotes in quotation marks "like this" to indicate direct quotes from your manuscript), or elaborate on this item by providing additional information not in the ms, or briefly explain why the item is not applicable/relevant for your study

"Previous studies reported effect sizes are mostly greater than one[20,21], prompting our use of Cohen's d=0.8. With α=0.05, power=0.90, ICC=0.05[22], and 1:1 allocation, the required sample size was 40 per group. Accounting for a 30% attrition rate, the total target sample was 116. We initially enrolled 120 PWD caregivers via cluster randomization (60 intervention, 60 control). During the trial, 9 intervention and 5 control participants withdrew, leaving 51 and 55 completers, respectively. "

7b) When applicable, explanation of any interim analyses and stopping guidelines

Does your paper address CONSORT subitem 7b? *

Copy and paste relevant sections from the manuscript (include quotes in quotation marks "like this" to indicate direct quotes from your manuscript), or elaborate on this item by providing additional information not in the ms, or briefly explain why the item is not applicable/relevant for your study

NA. "Data collection was conducted at two time points: the baseline survey (collecting both basic information and outcome measures) and the final survey (focusing solely on outcome measures)"

8a) Method used to generate the random allocation sequence

NPT: When applicable, how care providers were allocated to each trial group

Does your paper address CONSORT subitem 8a? *

Copy and paste relevant sections from the manuscript (include quotes in quotation marks "like this" to indicate direct quotes from your manuscript), or elaborate on this item by providing additional information not in the ms, or briefly explain why the item is not applicable/relevant for your study

"Family doctor teams served as key intervention implementers in China's primary healthcare system. Given their direct caregiver engagement and service delivery role, we randomized at the team level (cluster) to ensure implementation consistency. Using a computer–generated random sequence, an independent researcher allocated teams to intervention or control arms while maintaining allocation concealment. This approach preserved natural care delivery workflows while preventing selection bias, adhering to cluster randomized controlled trial guidelines. "

8b) Type of randomisation; details of any restriction (such as blocking and block size)

Does your paper address CONSORT subitem 8b? *

Copy and paste relevant sections from the manuscript (include quotes in quotation marks "like this" to indicate direct quotes from your manuscript), or elaborate on this item by providing additional information not in the ms, or briefly explain why the item is not applicable/relevant for your study

"Family doctor teams served as key intervention implementers in China's primary healthcare system. Given their direct caregiver engagement and service delivery role, we randomized at the team level (cluster) to ensure implementation consistency. Using a computer–generated random sequence, an independent researcher allocated teams to intervention or control arms while maintaining allocation concealment. This approach preserved natural care delivery workflows while preventing selection bias, adhering to cluster randomized controlled trial guidelines. "

9) Mechanism used to implement the random allocation sequence (such as sequentially numbered containers), describing any steps taken to conceal the sequence until interventions were assigned

Does your paper address CONSORT subitem 9? *

Copy and paste relevant sections from the manuscript (include quotes in quotation marks "like this" to indicate direct quotes from your manuscript), or elaborate on this item by providing additional information not in the ms, or briefly explain why the item is not applicable/relevant for your study

"Family doctor teams served as key intervention implementers in China's primary healthcare system. Given their direct caregiver engagement and service delivery role, we randomized at the team level (cluster) to ensure implementation consistency. Using a computer–generated random sequence, an independent researcher allocated teams to intervention or control arms while maintaining allocation concealment. This approach preserved natural care delivery workflows while preventing selection bias, adhering to cluster randomized controlled trial guidelines. "

10) Who generated the random allocation sequence, who enrolled participants, and who assigned participants to interventions

Does your paper address CONSORT subitem 10? *

Copy and paste relevant sections from the manuscript (include quotes in quotation marks "like this" to indicate direct quotes from your manuscript), or elaborate on this item by providing additional information not in the ms, or briefly explain why the item is not applicable/relevant for your study

"Participant recruitment and randomization
Community staff assisted with participant recruitment by providing access to the Civil Affairs Department's disability registry and resident health records. Researchers then verified eligibility based on the inclusion/exclusion criteria. Eligible participants were sequentially assigned in groups of 10 based on enrollment order, with each cluster assigned to a family doctor team for health management. The study participants were recruited between May and July 2024.

Family doctor teams served as key intervention implementers in China's primary healthcare system. Given their direct caregiver engagement and service delivery role, we randomized at the team level (cluster) to ensure implementation consistency. Using a computer–generated random sequence, an independent researcher allocated teams to intervention or control arms while maintaining allocation concealment. This approach preserved natural care delivery workflows while preventing selection bias, adhering to cluster randomized controlled trial guidelines. "

11a) If done, who was blinded after assignment to interventions (for example, participants, care providers, those assessing outcomes) and how

NPT: Whether or not administering co-interventions were blinded to group assignment

11a-i) Specify who was blinded, and who wasn’t

Specify who was blinded, and who wasn’t. Usually, in web-based trials it is not possible to blind the participants [1, 3] (this should be clearly acknowledged), but it may be possible to blind outcome assessors, those doing data analysis or those administering co-interventions (if any).

	1	2	3	4	5	
subitem not at all important	○	○	○	○	○	essential

Does your paper address subitem 11a-i? *

Copy and paste relevant sections from the manuscript (include quotes in quotation marks "like this" to indicate direct quotes from your manuscript), or elaborate on this item by providing additional information not in the ms, or briefly explain why the item is not applicable/relevant for your study

Intervention
In China's primary healthcare system, a standard family doctor team consists of three core members: a general practitioner (family doctor), a nurse, and a public health worker. These teams deliver essential public health services for older adults through community health centers or township health centers, including routine health examinations, health management programs, and chronic disease management (primarily targeting hypertension and diabetes). These services constitute the fundamental components of conventional family doctor contract service (usual care).

In our intervention protocol, the intervention group received enhanced family doctor contract service incorporating iSupport–based dementia caregiver support. This augmented care model included all standard services plus proactive biweekly follow–ups conducted through WeChat or telephone, with three specific objectives: (1) ensuring timely completion of iSupport educational modules through structured reminders; (2) monitoring learning progression while addressing both course–related questions and practical caregiving difficulties; and (3) delivering psychosocial support to enhance engagement, including professional guidance for course content and caregiving challenges. To optimize communication, dedicated WeChat groups connected caregivers with their family doctor teams, enabling immediate problem–solving throughout the intervention. The control group continued receiving usual care without proactive engagement. Their family doctor teams maintained a responsive (rather than proactive) approach, addressing caregiver inquiries only when specifically requested, thereby preserving naturalistic care delivery conditions. Consistent with the 3–6 month intervention durations reported in prior studies and constrained by practical considerations, we implemented a 20–week intervention period. The intervention implementation process is presented in Figure 2.

11a-ii) Discuss e.g., whether participants knew which intervention was the "intervention of interest" and which one was the "comparator"

Informed consent procedures (4a-ii) can create biases and certain expectations - discuss e.g., whether participants knew which intervention was the "intervention of interest" and which one was the "comparator".

	1	2	3	4	5	
subitem not at all important	○	○	○	○	○	essential

Does your paper address subitem 11a-ii?

Copy and paste relevant sections from the manuscript (include quotes in quotation marks "like this" to indicate direct quotes from your manuscript), or elaborate on this item by providing additional information not in the ms, or briefly explain why the item is not applicable/relevant for your study

您的回答

11b) If relevant, description of the similarity of interventions

(this item is usually not relevant for ehealth trials as it refers to similarity of a placebo or sham intervention to a active medication/intervention)

Does your paper address CONSORT subitem 11b? *

Copy and paste relevant sections from the manuscript (include quotes in quotation marks "like this" to indicate direct quotes from your manuscript), or elaborate on this item by providing additional information not in the ms, or briefly explain why the item is not applicable/relevant for your study

"The control group continued receiving usual care without proactive engagement. Their family doctor teams maintained a responsive (rather than proactive) approach, addressing caregiver inquiries only when specifically requested, thereby preserving naturalistic care delivery conditions. "

12a) Statistical methods used to compare groups for primary and secondary outcomes

NPT: When applicable, details of whether and how the clustering by care providers or centers was addressed

Does your paper address CONSORT subitem 12a? *

Copy and paste relevant sections from the manuscript (include quotes in quotation marks "like this" to indicate direct quotes from your manuscript), or elaborate on this item by providing additional information not in the ms, or briefly explain why the item is not applicable/relevant for your study

"Data analysis
Intention–to–treat analysis was applied. Parametric continuous data were analyzed by t–tests with mean (SD); non–parametric data (median [IQR]) by Mann–Whitney U tests. Categorical data (n, %) used χ^2 tests. Two–tailed tests (α=0.05) reported exact P–values (except P<.001). For normally–distributed continuous outcomes (caregiver burden, health–related quality of life, social support) with hierarchical longitudinal structure, we employed linear mixed–effects models (LMM). Learning behavior data (overdispersed count variables) were analyzed using negative binomial generalized linear mixed models (GLMMs), with negative binomial distribution and log–link function were implemented."

12a-i) Imputation techniques to deal with attrition / missing values

Imputation techniques to deal with attrition / missing values: Not all participants will use the intervention/comparator as intended and attrition is typically high in ehealth trials. Specify how participants who did not use the application or dropped out from the trial were treated in the statistical analysis (a complete case analysis is strongly discouraged, and simple imputation techniques such as LOCF may also be problematic [4]).

	1	2	3	4	5	
subitem not at all important	○	○	○	◉	○	essential

清除所选内容

Does your paper address subitem 12a-i? *

Copy and paste relevant sections from the manuscript (include quotes in quotation marks "like this" to indicate direct quotes from your manuscript), or elaborate on this item by providing additional information not in the ms, or briefly explain why the item is not applicable/relevant for your study

"Intention–to–treat analysis was applied."

12b) Methods for additional analyses, such as subgroup analyses and adjusted analyses

Does your paper address CONSORT subitem 12b? *

Copy and paste relevant sections from the manuscript (include quotes in quotation marks "like this" to indicate direct quotes from your manuscript), or elaborate on this item by providing additional information not in the ms, or briefly explain why the item is not applicable/relevant for your study

No subgroup analysis. Both the descriptive statistics and regression results after controlling participants' demographic characteristics were presented.

X26) REB/IRB Approval and Ethical Considerations [recommended as subheading under "Methods"] (not a CONSORT item)

X26-i) Comment on ethics committee approval

	1	2	3	4	5	
subitem not at all important	○	○	○	○	●	essential

清除所选内容

Does your paper address subitem X26-i?

Copy and paste relevant sections from the manuscript (include quotes in quotation marks "like this" to indicate direct quotes from your manuscript), or elaborate on this item by providing additional information not in the ms, or briefly explain why the item is not applicable/relevant for your study

"Ethical considerations
The study was approved by the Ethics Committee of the Institute of Medical Information on November 28, 2023 (No. IMICAMS/05/23/HREC)."

x26-ii) Outline informed consent procedures

Outline informed consent procedures e.g., if consent was obtained offline or online (how? Checkbox, etc.?), and what information was provided (see 4a-ii). See [6] for some items to be included in informed consent documents.

	1	2	3	4	5	
subitem not at all important	○	○	○	◉	○	essential

清除所选内容

Does your paper address subitem X26-ii?

Copy and paste relevant sections from the manuscript (include quotes in quotation marks "like this" to indicate direct quotes from your manuscript), or elaborate on this item by providing additional information not in the ms, or briefly explain why the item is not applicable/relevant for your study

"During recruitment, researchers distributed informed consent forms to participants to ensure that they were fully aware of the study. Caregivers who decide to participate were clearly informed of their rights by the researchers. Their participation in this study was voluntary and they had the right to leave at any time and without reason, and that this decision did not have any adverse effect on them or the person they care for. They also had the right to refuse to answer questions if they feel uncomfortable. "

X26-iii) Safety and security procedures

Safety and security procedures, incl. privacy considerations, and any steps taken to reduce the likelihood or detection of harm (e.g., education and training, availability of a hotline)

	1	2	3	4	5	
subitem not at all important	○	○	○	○	○	essential

Does your paper address subitem X26-iii?

Copy and paste relevant sections from the manuscript (include quotes in quotation marks "like this" to indicate direct quotes from your manuscript), or elaborate on this item by providing additional information not in the ms, or briefly explain why the item is not applicable/relevant for your study

It is not applicable for this study.

RESULTS

13a) For each group, the numbers of participants who were randomly assigned, received intended treatment, and were analysed for the primary outcome

NPT: The number of care providers or centers performing the intervention in each group and the number of patients treated by each care provider in each center

Does your paper address CONSORT subitem 13a? *

Copy and paste relevant sections from the manuscript (include quotes in quotation marks "like this" to indicate direct quotes from your manuscript), or elaborate on this item by providing additional information not in the ms, or briefly explain why the item is not applicable/relevant for your study

The number of participants could be seen at "Table 1 Summary statistics of trial participants (n, %)".

13b) For each group, losses and exclusions after randomisation, together with reasons

Does your paper address CONSORT subitem 13b? (NOTE: Preferably, this is shown in a CONSORT flow diagram) *

Copy and paste relevant sections from the manuscript (include quotes in quotation marks "like this" to indicate direct quotes from your manuscript), or elaborate on this item by providing additional information not in the ms, or briefly explain why the item is not applicable/relevant for your study

"During the trial, 9 intervention and 5 control participants withdrew, leaving 51 and 55 completers, respectively. " More information were presented in Figure 2.

13b-i) Attrition diagram

Strongly recommended: An attrition diagram (e.g., proportion of participants still logging in or using the intervention/comparator in each group plotted over time, similar to a survival curve) or other figures or tables demonstrating usage/dose/engagement.

	1	2	3	4	5	
subitem not at all important	○	○	●	○	○	essential

清除所选内容

Does your paper address subitem 13b-i?

Copy and paste relevant sections from the manuscript or cite the figure number if applicable (include quotes in quotation marks "like this" to indicate direct quotes from your manuscript), or elaborate on this item by providing additional information not in the ms, or briefly explain why the item is not applicable/relevant for your study

您的回答

14a) Dates defining the periods of recruitment and follow-up

Does your paper address CONSORT subitem 14a? *

Copy and paste relevant sections from the manuscript (include quotes in quotation marks "like this" to indicate direct quotes from your manuscript), or elaborate on this item by providing additional information not in the ms, or briefly explain why the item is not applicable/relevant for your study

"The study participants were recruited between May and July 2024." "Data collection was conducted at two time points: the baseline survey (collecting both basic information and outcome measures) and the final survey (focusing solely on outcome measures)."

14a-i) Indicate if critical “secular events” fell into the study period

Indicate if critical “secular events” fell into the study period, e.g., significant changes in Internet resources available or “changes in computer hardware or Internet delivery resources”

	1	2	3	4	5	
subitem not at all important	○	○	◉	○	○	essential

清除所选内容

Does your paper address subitem 14a-i?

Copy and paste relevant sections from the manuscript (include quotes in quotation marks "like this" to indicate direct quotes from your manuscript), or elaborate on this item by providing additional information not in the ms, or briefly explain why the item is not applicable/relevant for your study

您的回答

14b) Why the trial ended or was stopped (early)

Does your paper address CONSORT subitem 14b? *

Copy and paste relevant sections from the manuscript (include quotes in quotation marks "like this" to indicate direct quotes from your manuscript), or elaborate on this item by providing additional information not in the ms, or briefly explain why the item is not applicable/relevant for your study

"Consistent with the 3–6 month intervention durations reported in prior studies and constrained by practical considerations, we implemented a 20–week intervention period."

15) A table showing baseline demographic and clinical characteristics for each group

NPT: When applicable, a description of care providers (case volume, qualification, expertise, etc.) and centers (volume) in each group

Does your paper address CONSORT subitem 15? *

Copy and paste relevant sections from the manuscript (include quotes in quotation marks "like this" to indicate direct quotes from your manuscript), or elaborate on this item by providing additional information not in the ms, or briefly explain why the item is not applicable/relevant for your study

"Baseline Characteristics of Trial Participants
As shown in Table 1, the intervention and control groups demonstrated balanced baseline characteristics across most measured variables (all P >.05), with the exception of caregiver age, where a statistically significant between–group difference was observed (P<.05). This finding suggests that randomization successfully achieved comparable groups for all key demographic and clinical variables except for caregiver age, which will be accounted for in subsequent adjusted analyses. " More information was presented in Table 1.

15-i) Report demographics associated with digital divide issues

In ehealth trials it is particularly important to report demographics associated with digital divide issues, such as age, education, gender, social-economic status, computer/Internet/ehealth literacy of the participants, if known.

	1	2	3	4	5	
subitem not at all important	○	○	◉	○	○	essential

清除所选内容

Does your paper address subitem 15-i? *

Copy and paste relevant sections from the manuscript (include quotes in quotation marks "like this" to indicate direct quotes from your manuscript), or elaborate on this item by providing additional information not in the ms, or briefly explain why the item is not applicable/relevant for your study

More information was presented in Table 1.

16) For each group, number of participants (denominator) included in each analysis and whether the analysis was by original assigned groups

16-i) Report multiple “denominators” and provide definitions

Report multiple “denominators” and provide definitions: Report N’s (and effect sizes) “across a range of study participation [and use] thresholds” [1], e.g., N exposed, N consented, N used more than x times, N used more than y weeks, N participants “used” the intervention/comparator at specific pre-defined time points of interest (in absolute and relative numbers per group). Always clearly define “use” of the intervention.

	1	2	3	4	5	
subitem not at all important	○	○	○	○	○	essential

Does your paper address subitem 16-i? *

Copy and paste relevant sections from the manuscript (include quotes in quotation marks "like this" to indicate direct quotes from your manuscript), or elaborate on this item by providing additional information not in the ms, or briefly explain why the item is not applicable/relevant for your study

The sample was 60 for both the intervention group and control group. The were clearly showed in the Table 1.

16-ii) Primary analysis should be intent-to-treat

Primary analysis should be intent-to-treat, secondary analyses could include comparing only "users", with the appropriate caveats that this is no longer a randomized sample (see 18-i).

	1	2	3	4	5	
subitem not at all important	○	○	○	◉	○	essential

清除所选内容

Does your paper address subitem 16-ii?

Copy and paste relevant sections from the manuscript (include quotes in quotation marks "like this" to indicate direct quotes from your manuscript), or elaborate on this item by providing additional information not in the ms, or briefly explain why the item is not applicable/relevant for your study

"Intention–to–treat analysis was applied."

17a) For each primary and secondary outcome, results for each group, and the estimated effect size and its precision (such as 95% confidence interval)

Does your paper address CONSORT subitem 17a? *

Copy and paste relevant sections from the manuscript (include quotes in quotation marks "like this" to indicate direct quotes from your manuscript), or elaborate on this item by providing additional information not in the ms, or briefly explain why the item is not applicable/relevant for your study

Detailed information was shown in Table 2.

17a-i) Presentation of process outcomes such as metrics of use and intensity of use

In addition to primary/secondary (clinical) outcomes, the presentation of process outcomes such as metrics of use and intensity of use (dose, exposure) and their operational definitions is critical. This does not only refer to metrics of attrition (13-b) (often a binary variable), but also to more continuous exposure metrics such as "average session length". These must be accompanied by a technical description how a metric like a "session" is defined (e.g., timeout after idle time) [1] (report under item 6a).

	1	2	3	4	5	
subitem not at all important	○	○	○	○	○	essential

Does your paper address subitem 17a-i?

Copy and paste relevant sections from the manuscript (include quotes in quotation marks "like this" to indicate direct quotes from your manuscript), or elaborate on this item by providing additional information not in the ms, or briefly explain why the item is not applicable/relevant for your study

您的回答

17b) For binary outcomes, presentation of both absolute and relative effect sizes is recommended

Does your paper address CONSORT subitem 17b? *

Copy and paste relevant sections from the manuscript (include quotes in quotation marks "like this" to indicate direct quotes from your manuscript), or elaborate on this item by providing additional information not in the ms, or briefly explain why the item is not applicable/relevant for your study

Detailed information was shown in Table 2.

18) Results of any other analyses performed, including subgroup analyses and adjusted analyses, distinguishing pre-specified from exploratory

Does your paper address CONSORT subitem 18? *

Copy and paste relevant sections from the manuscript (include quotes in quotation marks "like this" to indicate direct quotes from your manuscript), or elaborate on this item by providing additional information not in the ms, or briefly explain why the item is not applicable/relevant for your study

No subgroup analysis. We conducted descriptive statistical analysis and regression analysis.

18-i) Subgroup analysis of comparing only users

A subgroup analysis of comparing only users is not uncommon in ehealth trials, but if done, it must be stressed that this is a self-selected sample and no longer an unbiased sample from a randomized trial (see 16-iii).

	1	2	3	4	5	
subitem not at all important	○	○	○	○	○	essential

Does your paper address subitem 18-i?

Copy and paste relevant sections from the manuscript (include quotes in quotation marks "like this" to indicate direct quotes from your manuscript), or elaborate on this item by providing additional information not in the ms, or briefly explain why the item is not applicable/relevant for your study

您的回答

19) All important harms or unintended effects in each group
(for specific guidance see CONSORT for harms)

Does your paper address CONSORT subitem 19? *

Copy and paste relevant sections from the manuscript (include quotes in quotation marks "like this" to indicate direct quotes from your manuscript), or elaborate on this item by providing additional information not in the ms, or briefly explain why the item is not applicable/relevant for your study

NA. iSupport web-based education program is recommended by WHO. There was no expected harms for the participants.

19-i) Include privacy breaches, technical problems

Include privacy breaches, technical problems. This does not only include physical "harm" to participants, but also incidents such as perceived or real privacy breaches [1], technical problems, and other unexpected/unintended incidents. "Unintended effects" also includes unintended positive effects [2].

	1	2	3	4	5	
subitem not at all important	○	○	○	○	○	essential

Does your paper address subitem 19-i?

Copy and paste relevant sections from the manuscript (include quotes in quotation marks "like this" to indicate direct quotes from your manuscript), or elaborate on this item by providing additional information not in the ms, or briefly explain why the item is not applicable/relevant for your study

Detailed in the inform consent during the recruitment stage.

19-ii) Include qualitative feedback from participants or observations from staff/researchers

Include qualitative feedback from participants or observations from staff/researchers, if available, on strengths and shortcomings of the application, especially if they point to unintended/unexpected effects or uses. This includes (if available) reasons for why people did or did not use the application as intended by the developers.

	1	2	3	4	5	
subitem not at all important	○	○	○	◉	○	essential

清除所选内容

Does your paper address subitem 19-ii?

Copy and paste relevant sections from the manuscript (include quotes in quotation marks "like this" to indicate direct quotes from your manuscript), or elaborate on this item by providing additional information not in the ms, or briefly explain why the item is not applicable/relevant for your study

Qualitative interviews were conducted with key stakeholders by using CFIR to analyze "Implementation determinants across CFIR domains"

DISCUSSION

22) Interpretation consistent with results, balancing benefits and harms, and considering other relevant evidence

NPT: In addition, take into account the choice of the comparator, lack of or partial blinding, and unequal expertise of care providers or centers in each group

22-i) Restate study questions and summarize the answers suggested by the data, starting with primary outcomes and process outcomes (use)

Restate study questions and summarize the answers suggested by the data, starting with primary outcomes and process outcomes (use).

	1	2	3	4	5	
subitem not at all important	○	○	○	◉	○	essential

清除所选内容

Does your paper address subitem 22-i? *

Copy and paste relevant sections from the manuscript (include quotes in quotation marks "like this" to indicate direct quotes from your manuscript), or elaborate on this item by providing additional information not in the ms, or briefly explain why the item is not applicable/relevant for your study

All the detailed information could be found in the "Discussion" part of Manuscript.

22-ii) Highlight unanswered new questions, suggest future research

Highlight unanswered new questions, suggest future research.

	1	2	3	4	5	
subitem not at all important	○	○	◉	○	○	essential

清除所选内容

Does your paper address subitem 22-ii?

Copy and paste relevant sections from the manuscript (include quotes in quotation marks "like this" to indicate direct quotes from your manuscript), or elaborate on this item by providing additional information not in the ms, or briefly explain why the item is not applicable/relevant for your study

All the detailed information could be found in the "Discussion" part of Manuscript.
"Lessons learned and policy implications
This study highlights four essential strategies for effective dementia caregiver interventions: (1) Policy integration: Formally include dementia care in public health programs with dedicated funding. Implement performance incentives for primary healthcare providers and coordinate across health and social services. Mandate standardized training for physicians. (2) Adaptive digital solutions: Develop age–friendly platforms (WeChat mini–programs, voice–guided tools) with bite–sized content (3–5 minute videos). Distribute complementary physical materials through family doctor teams to ensure broad accessibility and higher engagement. (3) Tailored support systems: Customize interventions by caregiver needs, such as simplified modules for overwhelmed caregivers, analog options for tech beginners, and advanced resources for engaged learners. Integrate mandatory mental health support. (4) Comprehensive support networks: Build connections between family doctors, peer groups, and mental health services. Implement regular screenings, organized peer support, and community partnerships for sustainable care."

20) Trial limitations, addressing sources of potential bias, imprecision, and, if relevant, multiplicity of analyses

20-i) Typical limitations in ehealth trials

Typical limitations in ehealth trials: Participants in ehealth trials are rarely blinded. Ehealth trials often look at a multiplicity of outcomes, increasing risk for a Type I error. Discuss biases due to non-use of the intervention/usability issues, biases through informed consent procedures, unexpected events.

	1	2	3	4	5	
subitem not at all important	○	○	○	●	○	essential

清除所选内容

Does your paper address subitem 20-i? *

Copy and paste relevant sections from the manuscript (include quotes in quotation marks "like this" to indicate direct quotes from your manuscript), or elaborate on this item by providing additional information not in the ms, or briefly explain why the item is not applicable/relevant for your study

"However, while the online delivery model improved accessibility without substantially increasing primary healthcare providers’ workload, its pragmatic reliance on online delivery limited hands–on skill coaching and emotional support, potentially reducing the practical application of acquired knowledge in real caregiving situations. Variability in primary healthcare providers’ dementia expertise and supervisory approaches further contributed to inconsistent implementation fidelity. Although the 20–week intervention period followed common practice, the lack of long–term follow–up prevents assessment of sustained effects on caregiver burden, quality of life, and social support outcomes. These findings illustrate the fundamental challenge of balancing technological scalability with the relational demands of dementia care, suggesting future interventions might benefit from blended approaches that combine digital efficiency with essential human–centered elements."

21) Generalisability (external validity, applicability) of the trial findings

NPT: External validity of the trial findings according to the intervention, comparators, patients, and care providers or centers involved in the trial

21-i) Generalizability to other populations

Generalizability to other populations: In particular, discuss generalizability to a general Internet population, outside of a RCT setting, and general patient population, including applicability of the study results for other organizations

	1	2	3	4	5	
subitem not at all important	○	○	○	○	○	essential

Does your paper address subitem 21-i?

Copy and paste relevant sections from the manuscript (include quotes in quotation marks "like this" to indicate direct quotes from your manuscript), or elaborate on this item by providing additional information not in the ms, or briefly explain why the item is not applicable/relevant for your study

"Lessons learned and policy implications
This study highlights four essential strategies for effective dementia caregiver interventions: (1) Policy integration: Formally include dementia care in public health programs with dedicated funding. Implement performance incentives for primary healthcare providers and coordinate across health and social services. Mandate standardized training for physicians. (2) Adaptive digital solutions: Develop age–friendly platforms (WeChat mini–programs, voice–guided tools) with bite–sized content (3–5 minute videos). Distribute complementary physical materials through family doctor teams to ensure broad accessibility and higher engagement. (3) Tailored support systems: Customize interventions by caregiver needs, such as simplified modules for overwhelmed caregivers, analog options for tech beginners, and advanced resources for engaged learners. Integrate mandatory mental health support. (4) Comprehensive support networks: Build connections between family doctors, peer groups, and mental health services. Implement regular screenings, organized peer support, and community partnerships for sustainable care."

21-ii) Discuss if there were elements in the RCT that would be different in a routine application setting

Discuss if there were elements in the RCT that would be different in a routine application setting (e.g., prompts/reminders, more human involvement, training sessions or other co-interventions) and what impact the omission of these elements could have on use, adoption, or outcomes if the intervention is applied outside of a RCT setting.

	1	2	3	4	5	
subitem not at all important	○	○	○	◉	○	essential

清除所选内容

Does your paper address subitem 21-ii?

Copy and paste relevant sections from the manuscript (include quotes in quotation marks "like this" to indicate direct quotes from your manuscript), or elaborate on this item by providing additional information not in the ms, or briefly explain why the item is not applicable/relevant for your study

"Second, this study substantiates the inherent limitations of standalone digital interventions for dementia caregivers, demonstrating that purely online approaches yield suboptimal outcomes without integrated interpersonal support and cultural adaptation, as evidenced by the UK iSupport trial's minimal engagement (mean=4 logins/6 months) and null clinical effects[37]. Facilitated models exemplified by nurse–guided peer support achieved significant mental health improvements highlighting the critical need to address digital literacy disparities and psychosocial isolation among older caregivers[10].

While our intervention's biweekly physician follow–ups partially mitigated self–directed learning barriers, systemic limitations remained. Critically, the iSupport program's time–intensive design, particularly its reliance on slide–based and audio formats, proved fatiguing for older caregivers while yielding limited perceived benefits. Many participants viewed the program as burdensome, with qualitative feedback indicating a preference for respite services over educational content. These challenges highlight fundamental gaps that cannot be resolved through e–learning alone, necessitating policy–level interventions (such as mandated integration of caregiver support into primary care services) or structural support (such as development of community–based respite care networks)."

OTHER INFORMATION

23) Registration number and name of trial registry

Does your paper address CONSORT subitem 23? *

Copy and paste relevant sections from the manuscript (include quotes in quotation marks "like this" to indicate direct quotes from your manuscript), or elaborate on this item by providing additional information not in the ms, or briefly explain why the item is not applicable/relevant for your study

"Trial registration
The study was registered with the Chinese Clinical Trial Registry (Trial No. ChiCTR2400084788)."

24) Where the full trial protocol can be accessed, if available

Does your paper address CONSORT subitem 24? *

Cite a Multimedia Appendix, other reference, or copy and paste relevant sections from the manuscript (include quotes in quotation marks "like this" to indicate direct quotes from your manuscript), or elaborate on this item by providing additional information not in the ms, or briefly explain why the item is not applicable/relevant for your study

The protocol was not published. Visit trial registration to obtain more information. "The study was registered with the Chinese Clinical Trial Registry (Trial No. ChiCTR2400084788)."

25) Sources of funding and other support (such as supply of drugs), role of funders

Does your paper address CONSORT subitem 25? *

Copy and paste relevant sections from the manuscript (include quotes in quotation marks "like this" to indicate direct quotes from your manuscript), or elaborate on this item by providing additional information not in the ms, or briefly explain why the item is not applicable/relevant for your study

"This study is supported by Ministry of Education Humanities and Social Sciences Grant (22YJC630196), the National Natural Science Foundation of China (72304282) and Capital's Funds for Health Improvement and Research (CFH2024–2G–4311)."

X27) Conflicts of Interest (not a CONSORT item)

X27-i) State the relation of the study team towards the system being evaluated

In addition to the usual declaration of interests (financial or otherwise), also state the relation of the study team towards the system being evaluated, i.e., state if the authors/evaluators are distinct from or identical with the developers/sponsors of the intervention.

	1	2	3	4	5	
subitem not at all important	○	○	○	◉	○	essential

清除所选内容

Does your paper address subitem X27-i?

Copy and paste relevant sections from the manuscript (include quotes in quotation marks "like this" to indicate direct quotes from your manuscript), or elaborate on this item by providing additional information not in the ms, or briefly explain why the item is not applicable/relevant for your study

Conflicts of Interest: None declared.

About the CONSORT EHEALTH checklist

As a result of using this checklist, did you make changes in your manuscript? *

- ○ yes, major changes
- ◉ yes, minor changes
- ○ no

What were the most important changes you made as a result of using this checklist?

您的回答

How much time did you spend on going through the checklist INCLUDING making changes in your manuscript *

It took me almost two hours.

As a result of using this checklist, do you think your manuscript has improved? *

- (●) yes
- ( ) no
- ( ) 其他:

Would you like to become involved in the CONSORT EHEALTH group?

This would involve for example becoming involved in participating in a workshop and writing an "Explanation and Elaboration" document

- ( ) yes
- (●) no
- ( ) 其他:

清除所选内容

Any other comments or questions on CONSORT EHEALTH

您的回答

STOP - Save this form as PDF before you click submit

To generate a record that you filled in this form, we recommend to generate a PDF of this page (on a Mac, simply select "print" and then select "print as PDF") before you submit it.

When you submit your (revised) paper to JMIR, please upload the PDF as supplementary file.

Don't worry if some text in the textboxes is cut off, as we still have the complete information in our database. Thank you!

Final step: Click submit !

Click submit so we have your answers in our database!

提交 清除表单内容

切勿通过 Google 表单提交密码。

此内容不是由 Google 所创建，Google 不对其作任何担保。 - 服务条款 - 隐私权政策

此表单看起来很可疑吗？ 报告

Google 表单
